# Eudicot primary cell wall glucomannan is related in synthesis, structure, and function to xyloglucan

**DOI:** 10.1093/plcell/koac238

**Published:** 2022-08-04

**Authors:** Li Yu, Yoshihisa Yoshimi, Rosalie Cresswell, Raymond Wightman, Jan J Lyczakowski, Louis F L Wilson, Konan Ishida, Katherine Stott, Xiaolan Yu, Stephan Charalambous, Joel Wurman-Rodrich, Oliver M Terrett, Steven P Brown, Ray Dupree, Henry Temple, Kristian B R M Krogh, Paul Dupree

**Affiliations:** Department of Biochemistry, University of Cambridge, Hopkins Building, The Downing Site, Tennis Court Road, Cambridge CB2 1QW, UK; Department of Biochemistry, University of Cambridge, Hopkins Building, The Downing Site, Tennis Court Road, Cambridge CB2 1QW, UK; Department of Physics, University of Warwick, Coventry CV4 7AL, UK; Microscopy Core Facility, Sainsbury Laboratory, University of Cambridge, Bateman Street, Cambridge CB2 1LR, UK; Department of Biochemistry, University of Cambridge, Hopkins Building, The Downing Site, Tennis Court Road, Cambridge CB2 1QW, UK; Department of Biochemistry, University of Cambridge, Hopkins Building, The Downing Site, Tennis Court Road, Cambridge CB2 1QW, UK; Department of Biochemistry, University of Cambridge, Hopkins Building, The Downing Site, Tennis Court Road, Cambridge CB2 1QW, UK; Department of Biochemistry, University of Cambridge, Sanger Building , 80 Tennis Court Road, Cambridge CB2 1GA, UK; Department of Biochemistry, University of Cambridge, Hopkins Building, The Downing Site, Tennis Court Road, Cambridge CB2 1QW, UK; Department of Biochemistry, University of Cambridge, Hopkins Building, The Downing Site, Tennis Court Road, Cambridge CB2 1QW, UK; Department of Biochemistry, University of Cambridge, Hopkins Building, The Downing Site, Tennis Court Road, Cambridge CB2 1QW, UK; Department of Biochemistry, University of Cambridge, Hopkins Building, The Downing Site, Tennis Court Road, Cambridge CB2 1QW, UK; Department of Physics, University of Warwick, Coventry CV4 7AL, UK; Department of Physics, University of Warwick, Coventry CV4 7AL, UK; Department of Biochemistry, University of Cambridge, Hopkins Building, The Downing Site, Tennis Court Road, Cambridge CB2 1QW, UK; Novozymes A/S, Krogshøjvej 36, 2880 Bagsværd, Denmark; Department of Biochemistry, University of Cambridge, Hopkins Building, The Downing Site, Tennis Court Road, Cambridge CB2 1QW, UK

## Abstract

Hemicellulose polysaccharides influence assembly and properties of the plant primary cell wall (PCW), perhaps by interacting with cellulose to affect the deposition and bundling of cellulose fibrils. However, the functional differences between plant cell wall hemicelluloses such as glucomannan, xylan, and xyloglucan (XyG) remain unclear. As the most abundant hemicellulose, XyG is considered important in eudicot PCWs, but plants devoid of XyG show relatively mild phenotypes. We report here that a patterned β-galactoglucomannan (β-GGM) is widespread in eudicot PCWs and shows remarkable similarities to XyG. The sugar linkages forming the backbone and side chains of β-GGM are analogous to those that make up XyG, and moreover, these linkages are formed by glycosyltransferases from the same CAZy families. Solid-state nuclear magnetic resonance indicated that β-GGM shows low mobility in the cell wall, consistent with interaction with cellulose. Although Arabidopsis β-GGM synthesis mutants show no obvious growth defects, genetic crosses between β-GGM and XyG mutants produce exacerbated phenotypes compared with XyG mutants. These findings demonstrate a related role of these two similar but distinct classes of hemicelluloses in PCWs. This work opens avenues to study the roles of β-GGM and XyG in PCWs.

## Teaser

Patterned β-GGM resembles xyloglucan in structure, biosynthesis, and function.

In a Nutshell **Background:** Plant primary cell walls (PCWs) need to be rigid enough to define the plant shape and yet allow cell expansion at the same time. Plants achieve this by forming a complex network that is composed of cellulose and various non-cellulosic polysaccharides, such as hemicelluloses. Cell walls differ in the abundance of the various hemicelluloses, and their roles are poorly understood. In contrast to xyloglucan (XyG), which has been the most extensively studied hemicellulose in the PCWs, neither the structure nor functions of glucomannan has been resolved.

**Question:** Are the functions of the glucomannan in PCWs distinct from the roles of the most abundant hemicellulose, XyG?

**Findings:** We discovered a type of glucomannan in eudicot PCWs, which we named β-galactoglucomannan (β-GGM) because of its distinctive structures: disaccharide side chains of β-Gal-α-Gal and alternating repeats of Glc-Man in the backbone. Similarity to XyG in structure and biosynthesis led us to identify a β-galactosyltransferase for the β-GGM biosynthesis. We found that β-GGM contributed to normal cell expansion, in a way that was masked by the presence of XyG. These results suggest related functions of β-GGM to XyG, highlighting the necessity to consider the contribution of multiple hemicelluloses in the functional study of plant cell walls.

**Next steps:** We would like to know how β-GGM binds to cellulose, and how this differs to cellulose binding of XyG. Investigation of the precise arrangements and interactions of cellulose and hemicelluloses including β-GGM and XyG will help further understanding of the enigmatic functions of hemicelluloses.

## Introduction

Although the primary cell wall (PCW) is strong enough to protect the plant cell from osmotic lysis and to maintain cell and tissue shape, it can also allow the cell to expand irreversibly during growth. How the cell wall accommodates both these contrasting and fundamental properties is poorly understood. The PCW is a composite of relatively rigid cellulose microfibrils embedded in a highly hydrated matrix of non-cellulosic polysaccharides. The hemicellulose polysaccharides xyloglucan (XyG), xylan, and glucomannan can bind tightly to cellulose (Cavalier et al., 2008; Cosgrove, 2014; Simmons et al., 2016; Terrett et al., 2019). For many years, a cellulose-XyG network was proposed to be the principal load-bearing structure of the PCW in dicots (Cosgrove, 2018). However, experimental data are now more consistent with a view where cellulose fibril interactions largely determine wall extensibility (Zhang et al., 2021). Hemicelluloses such as XyG may influence cell wall extensibility by binding at potential localized sites of cellulose fibril interaction (hot spots) (Park and Cosgrove, 2015). Understanding how the different hemicelluloses contribute to plant cell wall assembly remains an important challenge in cell wall biology.

XyG is the best studied PCW hemicellulose and it has a repeating patterned structure. In most dicots, this unit is normally composed of four β-1,4-linked glucosyl (Glc) residues, with the first three backbone residues in each unit substituted with α-1,6-xylosyl (Xyl) branches. This unit can be conveniently described as “XXXG” (Supplemental Figure S1) using the established nomenclature (Fry et al., 1993). Xyl residues at positions 2 or 3 can be further decorated with β-1,2-galactose (Gal) (e.g. XXLG), galacturonic acid, or a variety of other sugars (Pauly and Keegstra, 2016), some of which may be further decorated with α-1,2-fucose. The XyG side chains probably influence the solubility of the polysaccharide during synthesis and secretion, as well as in the cell wall (Whitney et al., 2006; Han et al., 2020). The importance of the repeating structure of XyG is unclear, but it may influence how XyG adheres to surfaces of cellulose, impacting PCW properties (Zhao et al., 2014; Park and Cosgrove, 2015; Benselfelt et al., 2016). Indeed, the regular pattern of substitution of xylan, an unrelated hemicellulose, is thought to influence the binding of xylan to cellulose in secondary cell walls (SCWs) (Simmons et al., 2016; Grantham et al., 2017). The complete loss of XyG in the *Arabidopsis thaliana* XYLOGLUCAN XYLOSYLTRANSFERASE (XXT) double mutant *xxt1 xxt2* affects the production and arrangement of cellulose in PCW in hypocotyls (Xiao et al., 2016; Zhao et al., 2019). However, this XyG mutant, and the XyG-deficient quintuple *cellulose_synthase-like c* (*cslc*) backbone synthesis mutant, only show small perturbations in growth (Cavalier et al., 2008; Kim et al., 2020), raising questions about the importance of this hemicellulose in PCW. In contrast, the loss of MUR3-dependent β-1,2-galactosylation results in a “cabbage-like” rosette and dwarfed growth (Tamura et al., 2005; Tedman‐Jones et al., 2008). This reveals a specific and important role of the XyG disaccharide side chain (and its fucosylated derivative), which may maintain XyG solubility during secretion or assembly of the wall (Aryal et al., 2020; Velasquez et al., 2021).

In the glucomannan of SCWs, the backbone of β-1,4-linked mannosyl (Man) residues is randomly interspersed with β-1,4-Glc residues and sometimes bears occasional α-1,6-linked Gal branches. The Man residues are often acetylated; we refer here to this hemicellulose as acetylated galactoglucomannan (AcGGM) (Goubet et al., 2009; Scheller and Ulvskov, 2010; Rodríguez-Gacio et al., 2012). Such glucomannans are particularly abundant in gymnosperm SCWs, where they interact with cellulose (Terrett et al., 2019; Cresswell et al., 2021). However, in contrast to the random backbone of the AcGGM polymer, a glucomannan from Arabidopsis seed mucilage has been found to exhibit a repeating backbone of the disaccharide [4-Glc-β-1,4-Man-β-1,], with frequent α-1,6-Gal branches on the Man residues (Voiniciuc et al., 2015; Yu et al., 2018). A glucomannan with elements of this repeating backbone also has been reported from kiwifruit (*Actinidia deliciosa*) and *Nicotiana plumbaginifolia* cell cultures (Sims et al., 1997; Schröder et al., 2001), but the structure of PCW glucomannan is, in general, not well characterized.

Evidence for the importance of glucomannan in the PCW has been obtained from mannan biosynthesis mutants. The α-1,6-Gal substitutions on the glucomannan of Arabidopsis mucilage are added by MANNAN ALPHA GALACTOSYLTRANSFERASE 1 (MAGT1)/MUCILAGE-RELATED10 (MUCI10) in CAZy family GT34 (Voiniciuc et al., 2015; Yu et al., 2018). Mutants in this glucomannan galactosyltransferase show defective mucilage architecture and cellulose rays. CELLULOSE SYNTHASE-LIKE A (CSLA) enzymes from CAZy family GT2 synthesize the glucomannan backbone (Liepman et al., 2005, 2007). The mucilage glucomannan backbone is made by CSLA2, and *csla2* mutants also show defective mucilage architecture (Yu et al., 2014). Arabidopsis mutants in CSLA9, which is largely responsible for SCW glucomannan synthesis, show no obvious changes in wall properties (Goubet et al., 2009). However, the embryo lethality of the Arabidopsis *csla7* mutant suggests an important role of glucomannan, at least in embryonic PCWs (Goubet et al., 2003). Recently glucomannan has also been implicated in etiolated hypocotyl gravitropic bending, which involves asymmetric cell expansion (Somssich et al., 2021). In addition, an increase in glucomannan abundance was recently observed in *xxt1 xxt2* mutants (Sowinski et al., 2022), which led to the suggestion that other matrix polysaccharides may compensate for a lack of XyG. Despite examples that glucomannan is important in some instances, the role for PCW glucomannan in plant growth and development, and whether that role is related to that of other hemicelluloses, remains obscure.

Here, we investigate the structure, biosynthesis, and function of PCW glucomannan. We report that a novel type of mannan is widely present in eudicot PCWs, and we name it β-GGM. β-GGM has a repeating backbone structure with evenly spaced α-Gal substitutions, some of which are further substituted with β-1,2-Gal. We identify the biosynthetic machinery required to synthesize the backbone and side chains. β-GGM has many structural and biosynthetic similarities with XyG, and it may also share some functions with XyG in the PCW. These results demonstrate that distinct hemicelluloses can have associated functions and that a patterned PCW hemicellulose in addition to XyG may have importance for cell expansion and plant development.

## Results

### Two glucomannan types with distinct structures, synthesized by CSLA2 and CSLA9, are widely present in Arabidopsis PCW-rich tissues

**Figure 1:**
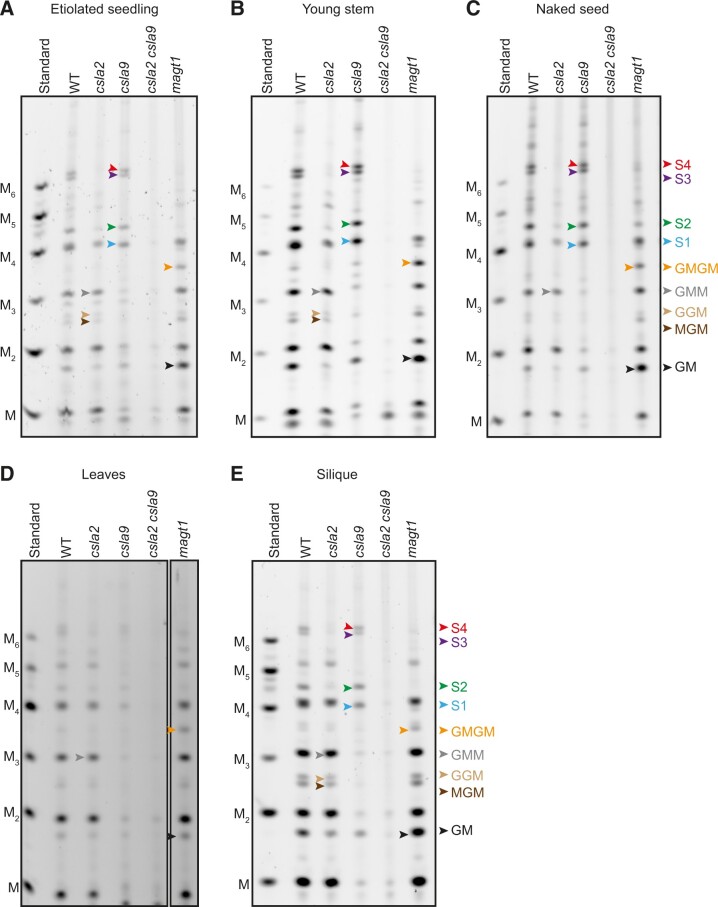
Two glucomannan types with distinct structures, synthesized by CSLA2 and CSLA9, are widely present in Arabidopsis PCW-rich tissues. Materials from five tissues (etiolated seedling, young stem, seeds with mucilage removed [naked seed], leaves, and silique) were analyzed by PACE. Hemicelluloses were extracted from Col-0, *csla2*, *csla9*, *csla2 csla9*, and *magt1* cell wall material using alkali before being hydrolyzed with *endo*-mannanase *Cj*Man26A. The products were subsequently derivatized with a fluorophore and separated by gel electrophoresis. The *csla2* mutant yielded oligosaccharides with a low DP, whereas the WT and *csla9* mutant walls yielded longer oligosaccharides. The four main oligosaccharides (named S1–S4) are labeled with colored arrows in samples from *csla9*. In leaves, the amount of S1–S4 was low, and they are missing in *magt1* mutants. M, Man; G, Glc; Manno-oligosaccharide standards M to M_6_ are shown.

We recently found that Arabidopsis mucilage glucomannan has a structure distinct from SCW acetylated glucomannan (AcGGM) (Yu et al., 2018). We therefore hypothesized that the fine structure of PCW glucomannan might also be distinct from SCW glucomannan. To investigate this, we digested alkali-extracted cell walls from etiolated Arabidopsis seedlings (which have relatively little tissue with SCW) with mannanase *Cj*Man26A, which cleaves GGM, yielding products with an unsubstituted Man residue at the reducing end (Gilbert, 2010; Yu et al., 2018). Using polysaccharide analysis by carbohydrate electrophoresis (PACE), we observed several different mannanase products (Figure 1A and Supplemental Figure S2). To determine the biosynthetic origin of these glucomannan fragments, we also analyzed cell wall material from *csla2* and *csla9* mutants. Digestion of the *csla2* mutant walls released mainly oligosaccharides with a low degree of polymerization (DP), whereas the *csla9* mutant walls yielded longer oligosaccharides, with four main oligosaccharides (named S1–S4). In contrast, mannanase digestion of the *csla2 csla9* double mutant walls released almost no detectable oligosaccharides. These results show that CSLA2 and CSLA9 are necessary for the synthesis of most *Cj*Man26A-digestible glucomannan in seedlings, and that each CSLA enzyme synthesizes glucomannans with distinct structures.

To investigate the mannan present in other PCW-rich tissues of Arabidopsis, alkali-extracted cell walls from young stem, seeds with mucilage removed (naked seeds), siliques, and leaves were also digested with *Cj*Man26A, and the released oligosaccharides visualized by PACE. The proportion of CSLA2- and CSLA9-dependent glucomannan oligosaccharides was similar in most of the tissues, and in each case, virtually no oligosaccharides were released from the *csla2 csla9* double mutant (Figure 1, A–E). However, in leaves, the CSLA9-dependent oligosaccharides were dominant, which suggests that CSLA9-dependent glucomannan can predominate in PCW in some tissues (Figure 1D). Together, our data indicate that two distinct glucomannans, with synthesis dependent on CSLA2 or CSLA9, are widely present in Arabidopsis PCW-rich tissues.

### β-GGM is a patterned glucomannan with similarities to XyG

**Figure 2:**
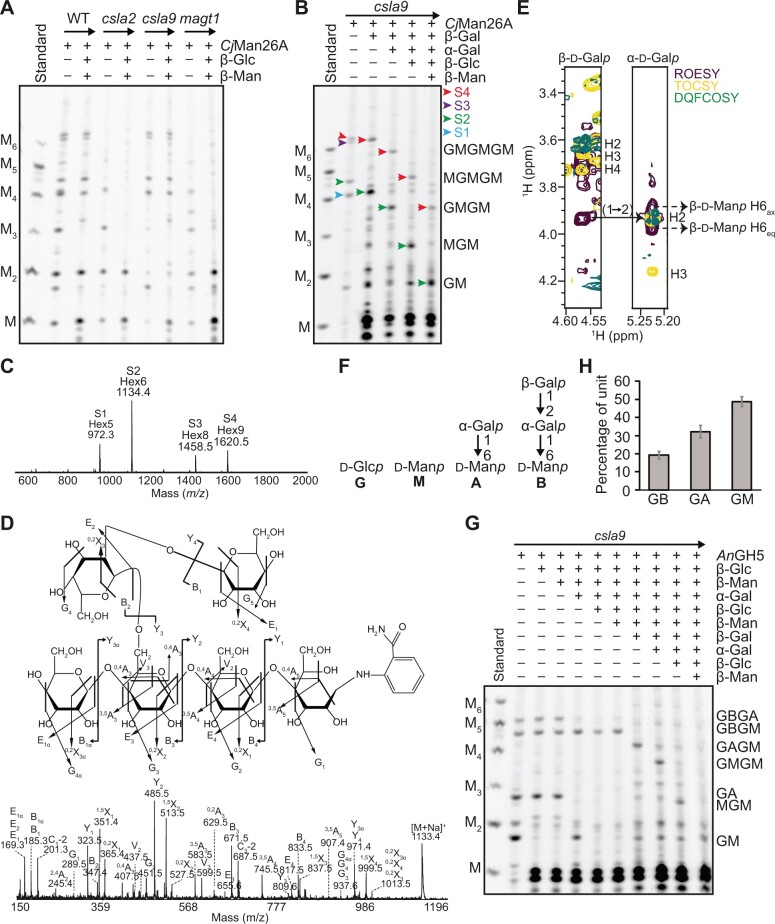
Structural analysis of β-galactosylated glucomannan oligosaccharides from Arabidopsis young stem. A, Characterization of glucomannan oligosaccharides released from WT, *csla2*, *csla9*, and *magt1* cell walls by *Cj*Man26A. Glucomannan from *csla2* is degraded into M, MM, GMM, and oligosaccharides migrating near M_4_. Many WT and *csla9* glucomannan oligosaccharides are resistant to β-glucosidase (β-Glc) and β-mannosidase (β-Man) enzyme digestions, whereas oligosaccharides from *csla2* are reduced to mono and disaccharides. B, Degradation of β-galactosylated glucomannan oligosaccharides from *csla9* young stem analyzed by PACE. β-Gal, α-Gal, β-Glc, and β-Man enzymes were used sequentially. C, Products of *Cj*Man26A digestion of *csla9* cell walls were labeled with 2-AB and analyzed by MALDI-TOF MS. The four main peaks correspond to the saccharides S1–S4. D, S2 Hex6 in C was analyzed by high-energy CID MS/MS. The CID spectrum indicates that the α-Gal residue is linked to C-6 of the third hexose from the reducing end and that the β-Gal residue is linked to the C-2 or C-3 of the α-Gal. E, Nuclear magnetic resonance (NMR) analysis of S2. H-1 strip plots from 2D ^1^H-^1^H TOSCY, ROESY, and DQFCOSY spectra, showing the nuclear Overhauser effect (NOE) connectivity arising from the β-Gal*p*-1,2-α-Gal*p* linkage. F, A single-letter nomenclature for the identified β-GGM backbone and possible side chains. G, Characterization of *An*GH5 β-GGM glucomannan digestion products by PACE. *An*GH5 cleaves β-GGM from *csla9* young stem cell walls into GM, GA, GBGM, and GBGA oligosaccharides. H, Proportion of β-GGM disaccharides with different side chains from *An*GH5 digestion of etiolated *csla9* seedling glucomannan and PACE densitometry (*n *=* *4). Error bars show the sd. Manno-oligosaccharide standards M to M_6_ are shown.

To determine the structures of the distinct glucomannan polysaccharides, we characterized the oligosaccharides released from the CSLA2- and CSLA9-dependent glucomannans. We focused first on the CSLA9-dependent oligosaccharides from *csla2* plants. From their migration in the PACE gel, we assigned the main *Cj*Man26A products as mannose, mannobiose, Glc-β-1,4-Man-β-1,4-Man (GMM, using a single letter code for each position), and Man-β-1,4-Glc-β-1,4-Man-β-1,4-Man (MGMM), consistent with a random distribution of Glc residues in the backbone—as reported in AcGGM from gymnosperm and angiosperm SCWs (Arnling Bååth et al., 2018). To help confirm these assignments, we treated the oligosaccharides with β-glucosidase and β-mannosidase, which can only fully depolymerize the backbone in the absence of α-Gal branches. PACE analysis of the products indicated that β-glucosidase and β-mannosidase could convert the CSLA9-dependent oligosaccharides to monosaccharides and disaccharides (Figure 2A). Hence, we could deduce that CSLA9-dependent glucomannan has very few of these α-Gal branches, and that the hemicellulose is not distinguishable from AcGGM reported from other plants.

Next, we analyzed the structure of CSLA2-dependent oligosaccharides released from *csla9* plants. We recently showed that the CSLA2-synthesized glucomannan in seed mucilage has a strictly repeating [4-Glc-β-1,4-Man-β-1,] disaccharide backbone with most of the Man residues substituted with α-1,6-Gal by the MAGT1 glycosyltransferase (Yu et al., 2018). Accordingly, to investigate if the oligosaccharides from etiolated seedlings were also α-galactosylated by MAGT1, we performed *Cj*Man26A digestions of *magt1* mutant seedling walls. The CSLA2-dependent oligosaccharides S1–S4 were absent or reduced in this mutant in all tissues, and two oligosaccharides corresponding to Glc-β-1,4-Man (GM) and Glc-β-1,4-Man-β-1,4-Glc-β-1,4-Man (GMGM) became more prominent (Figures 1 and 2A). Therefore, CSLA2 likely synthesizes a glucomannan with a repeating GM disaccharide backbone that is α-galactosylated by MAGT1.

To study the side chain structures in more detail, the four oligosaccharides S1–S4 were subjected to a sequential glycosidase digestion (Figure 2B). Since the presence of S1–S4 is dependent on MAGT1, we investigated whether they are sensitive to α-galactosidase treatment. Interestingly, only the mobilities of S1 and S3, but not S2 and S4, were altered by α-galactosidase (Supplemental Figure S3A). The two α-galactosidase-treated oligosaccharides could be fully hydrolyzed with alternating sequential β-glucosidase and β-mannosidase treatment, indicating that they are likely GMGM and GMGMGM. We analyzed all four oligosaccharides S1–S4 by matrix-assisted laser desorption/ionization time-of-flight (MALDI-ToF) mass spectrometry (MS). The resultant spectra presented four main ions corresponding to S1–S4, with mass Hex5 (*m/z* 972.3 [M+Na]^+^), Hex6 (*m/z* 1134.4 [M+Na]^+^), Hex8 (*m/z* 1458.5 [M+Na]^+^), and Hex9 (*m/z* 1620.5 [M+Na]^+^), respectively (Figure 2C). We reasoned that the mass of S1 and S3 likely corresponds to Hex5 and Hex8, and that they carry one and two α-Gal residues, respectively. Subsequent analysis of the S1 ion by collision-induced dissociation (CID) MS/MS located its α-Gal branch to the first Man residue from the non-reducing end in the GMGM structure (Supplemental Figure S3B). Combined with the fact that *Cj*Man26A requires an unsubstituted Man at the −1 subsite for hydrolysis, these PACE and MS results indicate that all Man residues in S1 and S3 except the reducing end are α-galactosylated.

Oligosaccharides S2 and S4 were resistant to all the above glycosidase treatments (Supplemental Figure S3A), suggesting that these oligosaccharides had additional terminal substitutions. In *N. plumbaginifolia* cell cultures and kiwifruit, a glucomannan with β-1,2-Gal decorations on its α-1,6-Gal residues has been identified (Sims et al., 1997; Schröder et al., 2001). Interestingly, after β-galactosidase treatment, S2 and S4 co-migrated with S1 and S3 (Figure 2B). Sequential digestion with α-galactosidase, β-glucosidase, and β-mannosidase confirmed that the β-galactosidase products had the same structure as S1 and S3. This indicates that S2 and S4 are S1 and S3 substituted with a β-Gal residue. Furthermore, CID MS/MS analysis of S2 showed that the second hexose from the reducing end is decorated with a hexose, which is itself substituted with a hexose, consistent with a β-Gal-α-Gal-disaccharide substitution of a backbone Man residue (Figure 2D). To confirm the linkage between the β-Gal and α-Gal, the S2 oligosaccharide was purified and analyzed by 2D nuclear magnetic resonance (NMR). ^1^H and ^13^C chemical-shift assignments are shown in Supplemental Table S1. The β-Gal residue was deduced to link to the α-Gal residue via a 1,2-linkage due to the downfield shift of the α-Gal C-2 and an intense ROE peak between β-Gal H-1 and α-Gal H-2 (Figure 2E). Therefore, CSLA2 synthesizes a glucomannan with a repeating GM disaccharide backbone, on which the Man residues may be decorated with either single α-1,6-Gal or a β-1,2-Gal-α-1,6-Gal disaccharide.

We named this glucomannan β-GGM because the β-Gal is one of the distinguishing features. By analogy to the XyG naming system, a one-letter code nomenclature was adopted to simplify the depiction of the arrangement of sugars and side chains along the backbone (Figure 2F). The letters G and M represent unsubstituted Glc and Man residues, respectively. α-1,6-Galactosylated Man residues are denoted by the letter A and the Man residues substituted by a Gal-β-1,2-Gal-α-1,6- disaccharide are denoted by the letter B. Using *An*GH5, which is a mannanase that can cleave following M or A units in a GGM backbone (von Freiesleben et al., 2016), digestion of β-GGM from the *csla9* young stem released four oligosaccharides (Figure 2G): GM, GA, GBGM, and GBGA. From these data, about 50% of backbone Man residues were decorated with α-1,6-Gal and about 40% of these α-Gal residues are further decorated with β-1,2-Gal (Figure 2H). Oligosaccharides with consecutive β-galactosylated Man residues were not seen (e.g. no GBGBGM, but GBGAGM and GBGM were seen), indicating that β-galactosylation is not random, but spaced at least four residues apart. Thus, in addition to the disaccharide backbone GM repeat, the β-GGM has a larger scale even-length pattern of at least four residues.

### β-GGM is widely present in eudicots

We considered whether β-GGM might be widespread in plants. As mentioned above, oligosaccharides that could arise from β-GGM were previously identified in *N. plumbaginifolia* cell cultures and kiwifruit samples (Sims et al., 1997; Schröder et al., 2001). We performed *An*GH5 mannanase digestions on alkali-extracted mannan from PCW-rich samples from tomato fruits, kiwi fruits, and apple fruits, representatives from the asterid and rosid eudicot clades. The β-GGM representative oligosaccharides GBGM and GAGM were present (Supplemental Figure S4, A–C). GBGM was digested by the β-galactosidase. Tomato fruit showed a higher proportion of GBGM oligosaccharide than the other plant tissues (Supplemental Figure S4), indicating that there is some variability in the level of β-Gal substitution of β-GGM.

We note that Arabidopsis seed mucilage glucomannan has a structure similar to β-GGM except that the β-Gal substitution was not reported (Yu et al., 2018). The MUM2 β-galactosidase is highly expressed in seed mucilage and has been shown to remove pectin terminal β-Gal (Dean et al., 2007; Macquet et al., 2007). We therefore considered the possibility that MUM2 also acts on β-GGM in mucilage to remove any β-Gal decoration. To test this hypothesis, alkali-extracted *mum2* mucilage was treated with *An*GH5 and analyzed by PACE. Minor GBGM and GBGA oligosaccharides were clearly present (Supplemental Figure S4D). We therefore conclude that mucilage mannan is also β-GGM and it has been partly trimmed by the MUM2 β-galactosidase. Arabidopsis mucilage glucomannan is not as unusual as previously thought (Yu et al., 2018), but another example of a tissue with β-GGM.

### AT4G13990 from GT47 clade A encodes β-GGM β-galactosyltransferase, MBGT1

**Figure 3:**
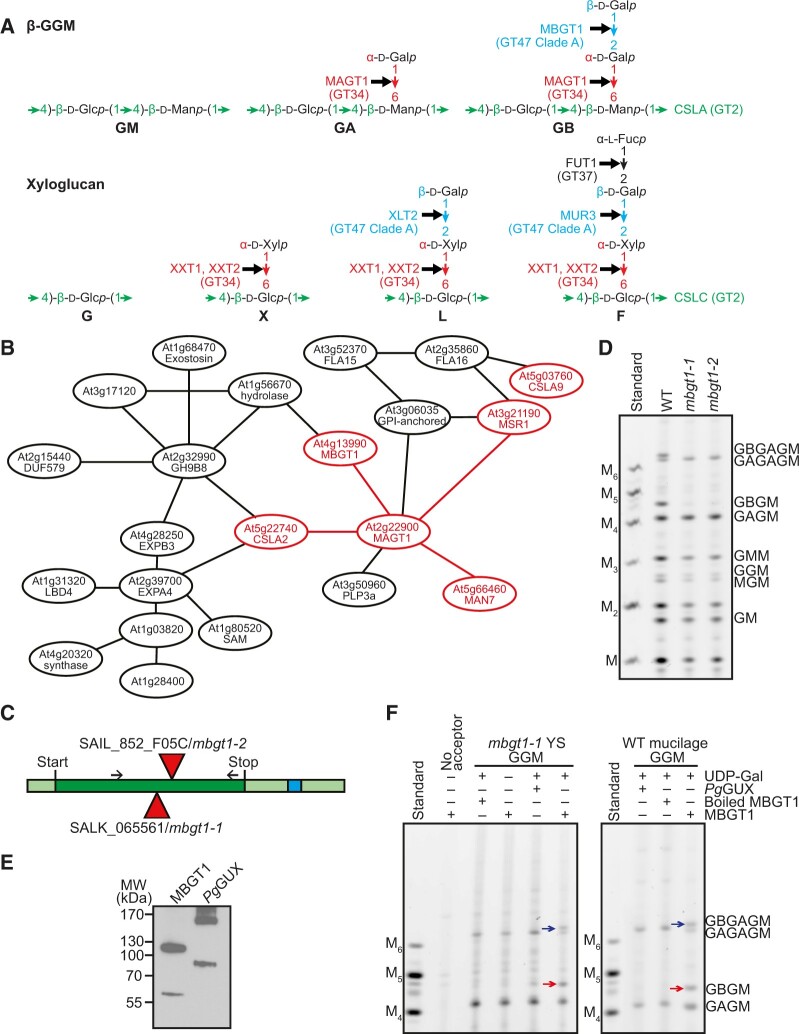
AT4G13990 from CAZy GT47 Clade A encodes Arabidopsis MBGT1. A, β-GGM and XyG share structural and biosynthesis similarities. These two polysaccharides exhibit analogous linkages in their backbones and corresponding side chain sugars. For each position in the hemicellulose, the responsible glycosyltransferases are from the same CAZy family. B, AT4G13990/MBGT1 from GT47 Clade A is in a co-expression network with CSLA2 and other mannan-related genes. C, Gene model representing *MBGT1*. Triangles represent the position of T-DNA insertions in mutant lines analyzed in this study. Dark green represents the exon. Light green represents the UTR and blue shows an intron. D, Stem material of two insertional mutants of the *MBGT1* gene was analyzed by PACE by *Cj*Man26A. No β-galactosylated oligosaccharide was detected in either *mbgt1* mutant. E, Immunoblot of 3× Myc-tagged recombinant proteins expressed in *N. benthamiana*. The expected mass of 3× Myc–MBGT1 is 64.86 kDa. The expected mass of the control enzyme 3× Myc–*Pg*GUX is 78.18 kDa. Data might suggest the proteins form stable dimers. F, In vitro activity of the recombinant MBGT1 protein. In the left panel, *mbgt1-1* young stem (YS) glucomannan was used as an acceptor for MBGT1-mediated galactosylation, whereas in the right panel, WT adherent mucilage glucomannan was used. The products were analyzed with PACE using digestion with *Cj*Man26A. Arrows indicate band shifts after each reaction. Manno-oligosaccharide standards M to M_6_ are shown.

To understand β-GGM biosynthesis, we attempted to identify the mannan β-galactosyltransferase (MBGT). We noted that β-GGM and XyG share high structural and biosynthetic similarities, summarized here and in Figure 3A. Both backbones have β-1,4-Glc residues in the backbone, which in β-GGM alternate with β-1,4-Man residues (Man differs from Glc only in epimerization of the C-2 OH). Both backbones are made by closely related GT2 members: the XyG backbone is synthesized by CSLCs (Cocuron et al., 2007; Liepman et al., 2007; Kim et al., 2020), while the β-GGM backbone is synthesized by a CSLA. Furthermore, the first side chain sugars are attached to the C-6 OH of Glc on the XyG backbone and to the C-6 OH of Man on the β-GGM backbone. The XyG α-1,6-Xyl is transferred by XXTs and α-1,6-Gal is transferred to β-GGM by MAGT1, both from the GT34 family (Scheller and Ulvskov, 2010). The disaccharide branch second sugar in β-GGM is β-1,2-Gal. The same sugar and linkage is found in XyG.

**Figure 4:**
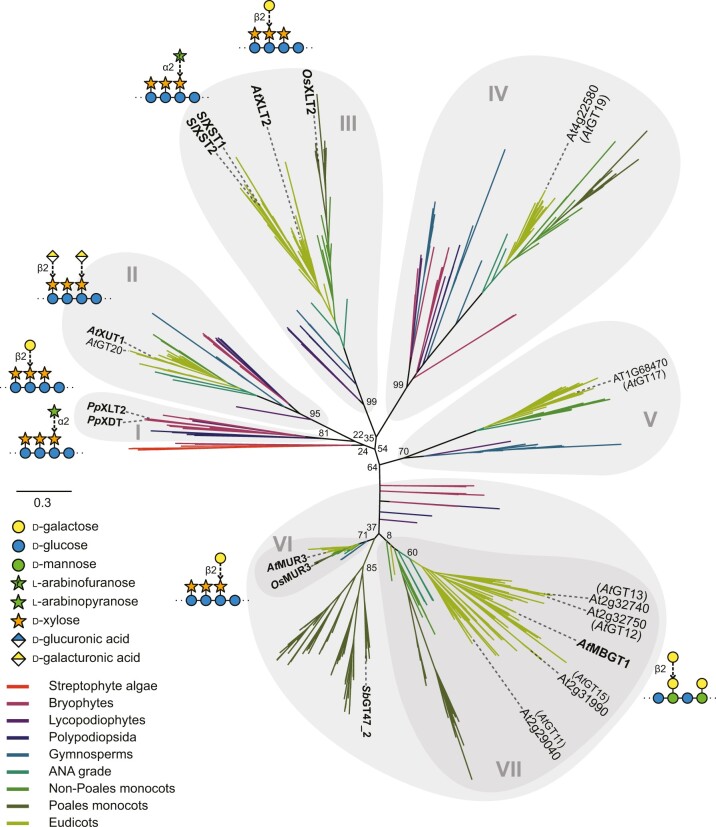
Un-rooted phylogenetic tree of CAZy GT47 Clade A. Sequences from the genomes of 96 streptophytes (Supplemental Table S2) were used to construct a comprehensive phylogeny of GT47 Clade A. Most sequences were downloaded from PLAZA (https://bioinformatics.psb.ugent.be/plaza/), but were supplemented with additional sequences from further genomes, derived from HMMER and TBLASTN searches. The streptophyte algae representative is *Klebsormidium nitens*, and the Lycopodiophyte representative is *Selaginella moellendorffii*. Sequences were aligned with MAFFT and truncated to leave only the predicted GT47 domain. The phylogeny was then inferred using FastTree, with 100 bootstrap pseudo-replicates. Percentage replication is indicated for important splits. Scale bar represents 0.3 substitutions per site. The resultant tree revealed the existence of seven main subgroups within GT47-A (groups I–VII), four of which contain known XyG glycosyltransferases. The group containing MBGT was designated group VII. For characterized enzymes, activities (as seen in Arabidopsis) are illustrated in SNFG format.

Given these extensive similarities between XyG and β-GGM, we hypothesized that MBGT might be found in GT47 clade A, which contains many XyG β-glycosyltransferases (MUR3, XLT2, and XUT1) (Geshi et al., 2018) and also many putative GTs with no known functions (classified *At*GT11-*At*GT20 in Li et al., 2004). To identify MBGT candidates, we constructed a comprehensive phylogeny of GT47-A sequences from across the plant kingdom. We collected GT47-A sequences from the genomes of 96 streptophytes (listed in Supplemental Table S2) and inferred an unrooted phylogeny (Figure 4). The sequences were clustered into at least seven groups: group I (containing only non-spermatophyte sequences, but including previously characterized *Pp*XLT2 and *Pp*XDT enzymes from *Physcomitrium patens* [Zhu et al., 2018]), group II (containing *At*XUT1 [Pena et al., 2012] and *At*GT20), group III (*At*XLT2 [Jensen et al., 2012], *Os*XLT2 from rice [*Oryza sativa*] [Liu et al., 2015], and tomato [*Solanum lycopersicum*] enzymes *Sl*XST1 and *Sl*XST2 [Schultink et al., 2013]), group IV (*At*GT19), group V (*At*GT17), group VI (*At*MUR3 [Madson et al., 2003], *Sl*MUR3 [Schultink et al., 2013], and *Os*MUR3 [Liu et al., 2015]), and group VII (*At*GT11–15).

Because none of the enzymes in groups IV, V, and VII had been characterized, we considered these groups to be a potential source of new activities (although *At*GT11 has recently been implicated in XyG synthesis in pollen tubes [Wei et al., 2021]). Accordingly, for each Arabidopsis gene within these GT47-A groups, we analyzed its co-expression using the co-expression database tool ATTED-II (Obayashi et al., 2018). Interestingly, we found that At4g13990 (*At*GT14, group VII) is co-expressed with the glucomannan biosynthetic enzymes CSLA2, MAGT1, and MSR1 (Figure 3B). Hence, we considered the possibility that At4g13990 could encode MBGT.

To assess the potential role of At4g13990/*At*GT14 in β-galactosylation of β-GGM, cell walls from young stems of two homozygous knockout At4g13990 lines (named *mbgt1-1* and *mbgt1-2*, Figure 3C) were digested with *Cj*Man26A and the products were analyzed by PACE. Remarkably, both mutant lines lacked the β-galactosylated β-GGM oligosaccharides (Figure 3D), indicating that this enzyme is required for normal β-galactosylation of β-GGM.

To confirm the activity of At4g13990/*At*GT14, we conducted an assay for MBGT activity in vitro using At4G13990 transiently expressed in *Nicotiana benthamiana* leaves. Alkali-treated cell wall materials from *mbgt1-1* young stem and WT adherent mucilage, rich in β-GGM but lacking β-galactosylation (Yu et al., 2018), were used as acceptors. To detect β-galactosylated glucomannan, the assay products were digested with mannanase *Cj*Man26A and analyzed by PACE. In the presence of UDP-Gal and microsomes from *N. benthamiana* expressing At4g13990/*At*GT14, β-GGM oligosaccharides were produced from mucilage and young stem acceptors (Figure 3, E and F). In contrast, when microsomes from *N. benthamiana* over-expressing *Picea glauca* GlucUronic acid substitution of Xylan (*Pg*GUX1) (Lyczakowski et al., 2017) were used as the control enzyme, no β-galactosylation was detected. Taken together with the mutant plants, these results confirm that At4g13990/*At*GT14 encodes MBGT, and so we named it MBGT1.

### Arabidopsis mutants in β-GGM and XyG side chain structure show negative genetic interactions

The structural and biosynthetic relationships between β-GGM and XyG suggest that these two polysaccharides may play related functions in vivo. If our hypothesis is correct, β-GGM biosynthesis disruption might exacerbate the phenotypes of XyG synthesis mutants.

**Figure 5:**
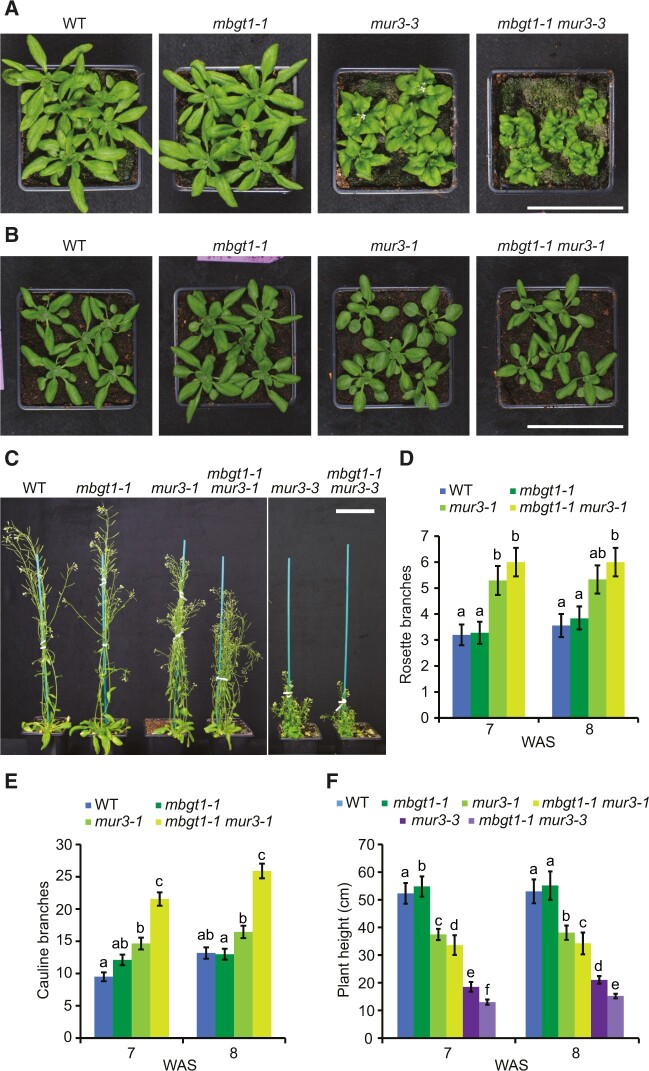
The importance of β-galactosylation of β-GGM is revealed in the XyG β-galactosylation mutant *mur3*. A, Four-week-old rosettes of *mur3-3* T-DNA insertion mutant and *mbgt1-1 mur3-3* double mutant. B, Four-week-old rosettes of *mur3-1* point mutant and *mbgt1-1 mur3-1* double mutant. C, Six-week-old plants, showing dwarfing of the *mur3* and *mbgt1-1 mur3* double mutants. D and E, Quantification of the number of rosette branches (D) and cauline branches (E) for 7- and 8-week-old *mur3-1* and *mbgt1-1 mur3-1* plants. *mbgt1-1 mur3-1* mutants show no significant change in rosette branches, but a significant increase in cauline branches compared with *mur3-1*. Data were modeled by Poisson regression; a likelihood ratio test indicated a significant contribution of genotype in determining the number of stems (Rosette branches 7 weeks: *n *=* *75, *G*^2^_3_ = 26.2, *P *=* *8.6 × 10^−6^; 8 weeks: *n *=* *74, *G*^2^_3_ = 16.6, *P *=* *8.4 × 10^−4^). Cauline branches 7 weeks: *n *=* *75, *G*^2^_3_ = 109, *P *<* *2.2 × 10^−16^; 8 weeks: *n *=* *73, *G*^2^_3_ = 144, *P *=* *1.5 × 10^−24^). Results of post hoc pairwise comparisons (within each time point) are indicated by compact letter display (letter sharing indicates lack of significant difference, i.e., where *P *>* *0.05). Data were modeled by Poisson regression; a likelihood ratio test indicated a significant contribution of genotype in determining the number of stems (Rosette branches 7 weeks: *n *=* *75, *G*^2^_3_ = 26.2, *P *=* *8.6 × 10^−6^; 8 weeks: *n *=* *74, *G*^2^_3_ = 16.6, *P *=* *8.4 × 10^−4^). Cauline branches 7 weeks: *n *=* *75, *G*^2^_3_ = 109, *P *<* *2.2 × 10^−16^; 8 weeks: *n *=* *73, *G*^2^_3_ = 144, *P *=* *1.5 × 10^−24^). Error bars represent standard error of the mean. F, Quantification of plant height for 7- and 8-week-old plants. One-way, two-tailed ANOVA indicated a significant contribution of genotype in determining plant height at both timepoints (7 weeks: *n *=* *208, *F*_5,202_ = 1257, *P *<* *2 × 10^−16^; 8 weeks: *n *=* *200, *F*_5,194_ = 760, *P *<* *2 × 10^−16^). Results of post hoc pairwise comparisons (within each time point) are indicated by compact letter display. Apart from the significant difference between WT and *mbgt1-*1 at 7 weeks, where *P *=* *0.0066, *P *<* *1 × 10^−6^ for all significant differences. Error bars represent standard deviation. WAS, week after sowing. Scale bars = 9 cm.

Mutant plants lacking β-GGM β-Gal (*mbgt1-1*) grew indistinguishably from wild-type plants (Figure 5). An analogous mutant in XyG is *mur3-3*, which lacks the third position β-Gal. It has a cabbage-like growth phenotype with curled rosette leaves, and short stems (Tamura et al., 2005; Tedman‐Jones et al., 2008). We generated *mbgt1-1 mur3-3* double mutant plants. As expected, they had no detectable β-GGM with B units and XyG with no third position L and F (β-Gal further substituted with Fuc) units (Supplemental Figure S5, A and B). Interestingly, these β-GGM and XyG double mutants had a smaller rosette than *mur3-3*, with more severely curled rosette leaves (Figure 5A). In addition, the inflorescence stem was shorter than the *mur3-3* single mutant plants (Figure 5C). The allelic Arabidopsis *mur3-1* mutant, with a single-point mutation in MUR3 (Madson et al., 2003; Jensen et al., 2012) also has defective XyG. For unclear reasons, this XyG mutant does not exhibit a cabbage phenotype, but the plants are shorter and have an increased number of rosette and cauline branches compared with WT (Jensen et al., 2012).

To test for genetic interactions with this allele, we generated *mbgt1-1 mur3-1* double mutant plants. Compared with the single mutant *mur3-1* plants, the *mbgt1-1 mur3-1* double mutant was significantly shorter and had more cauline branches (Figure 5, B–F). The increased severity of the *mur3-1* phenotypes when combined with *mbgt1-1* indicates that β-galactosylation of β-GMM is important for β-GMM function and suggests that the disaccharide side chains in both polysaccharides have similar functions.

### Arabidopsis mutants lacking β-GGM and XyG show negative genetic interactions

**Figure 6:**
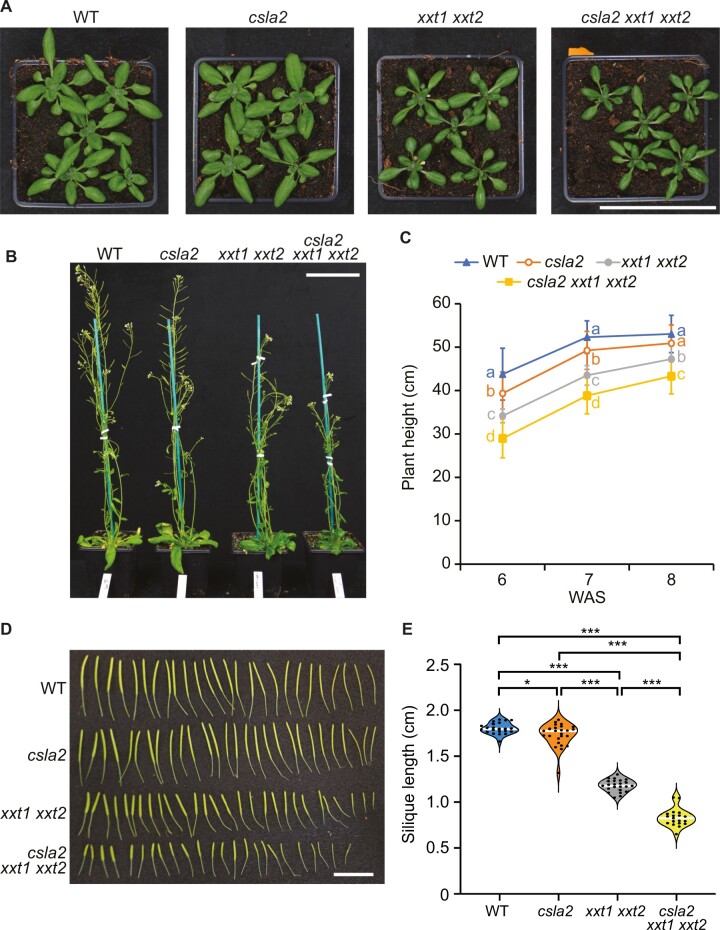
β-GGM function in PCWs is revealed when the XyG is missing. A, Four-week-old rosettes. Scale bar = 9 cm. B, Six-week-old plants. Scale bar = 9 cm. C, Quantification of plant height for 6-, 7-, and 8-week-old plants. One-way, two-tailed ANOVA indicated a significant contribution of genotype in determining plant height at all three timepoints (6 weeks: *n *=* *131, *F*_3,127_ = 65.0, *P *<* *2 × 10^−16^; 7 weeks: *n *=* *136, *F*_3,132_ = 88.2, *P *<* *2 × 10^−16^; 8 weeks: *n *=* *131, *F*_3,127_ = 35.8, *P *<* *2 × 10^−16^). Results of post hoc pairwise comparisons (Tukey’s honest significant difference) are indicated by compact letter display. For all significant differences, *P *<* *0.001 apart from WT–*clsa2* at week 7 (*P *=* *0.0063) and *csla2*–*xxt1 xxt2* at week 8 (*P *=* *0.0026). Error bars indicate standard deviation. D, Siliques from 7-week-old plants. Scale bar = 2 cm. E, Violin plot of silique length. Siliques from more than three plants were measured for each genotype. Black circles indicate individual measurements; white lines represent the group mean. One-way, two-tailed ANOVA indicated a significant contribution of genotype in determining silique length (*n *=* *89, *F*_3,85_ = 553, *P *<* *2 × 10^−16^). Results of post hoc pairwise comparisons (Tukey’s honest significant difference; WT, *n *=* *22; *csla2*, *n *=* *25; *xxt1 xxt2*, *n *=* *23; *csla2 xxt1 xxt2*, *n *=* *19) are indicated with asterisks (*P <* *0.05, ***P <* *0.001).

The *xxt1 xxt2* mutant, lacking detectable amounts of XyG, exhibits some morphological phenotypes in many tissues, yet the plants grow relatively normally. To investigate if the absence of β-GGM exacerbates the phenotype of these plants, we crossed the *csla2* mutant with *xxt1 xxt2*. As previously reported, compared with wild type, the *xxt1 xxt2* mutant had narrow leaves and a smaller rosette diameter, somewhat shorter plants at 8 weeks, and shorter siliques (Kong et al., 2015; Figure 6). The *csla2* mutant plants, lacking β-GGM, grew comparably to WT with marginal reductions in height and silique length. Interestingly, the *csla2 xxt1 xxt2* mutant plants, lacking both β-GGM and XyG (Supplemental Figure S5C), had a more severe phenotype with slightly changed rosette appearance, significantly shorter stems at 6–8 weeks and shorter siliques than *xxt1 xxt2* (Figure 6). Although it has been reported that *xxt1 xxt2* shows an increased amount of glucomannan (Sowinski et al., 2022), we detected no compensatory changes in the amounts of AcGGM and β-GGM in the mutants that we generated (Supplemental Figures S5 and S6).

**Figure 7:**
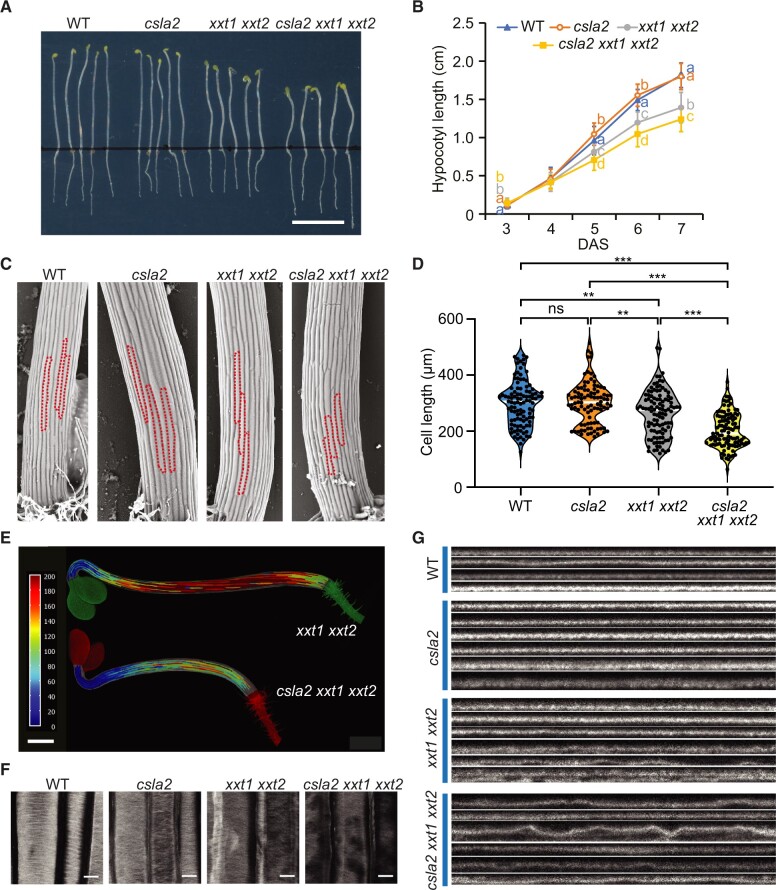
A role of β-GGM in cell expansion and cellulose organization. A, Six-day-old hypocotyls grown on MS medium with sucrose. Scale bar = 1 cm. B, Quantification of hypocotyl length for 3- to 6-day-old seedlings (*n* ≥ 40 seedlings for each point per genotype). DAS, days after sowing. Error bars represent standard deviation. Although one-way, two-tailed ANOVA indicated no significant difference between genotypes at 4 days (*n *=* *213, *F*_3,209_ = 2.58, *P *=* *0.054), a significant difference was seen at 3 days, 5 days, and after (3 days: *n *=* *197, *F*_3,193_ = 40.7, *P *<* *2 × 10^−16^; 5 days: *n *=* *276, *F*_3,272_ = 82.8, *P *<* *2 × 10^−16^; 6 days: *n *=* *271, *F*_3,267_ = 177, *P *<* *2 × 10^−16^; 7 days: *n *=* *245, *F*_3,241_ = 167, *P *<* *2 × 10^−16^). Results of post hoc pairwise comparisons (Tukey’s honest significant difference) are indicated by compact letter display. C, Cryo-SEM analysis of 4-day-old etiolated seedlings from WT and mutant plants. Individual cells in the tissue are outlined. Cells are shorter in the *csla2 xxt1 xxt2* triple mutant than in the *xxt1 xxt2* double mutant. Scale bar = 100 μm. D, Quantification of cell length of 4-day-old hypocotyls. Black circles indicate individual measurements; white lines represent the group mean. One-way, two-tailed ANOVA indicated a significant contribution of genotype in determining hypocotyl cell length (*n *=* *413, *F*_3,409_ = 40.44, *P *<* *2 × 10^−16^). Results of post hoc pairwise comparisons (Tukey’s honest significant difference) are indicated by asterisks (*P <* *0.05, **P <* *0.01, ***P <* *0.001). E, Heatmap showing 4-day-old hypocotyl cell length. Scale bar = 500 μm. F and G, Four-day-old hypocotyls were stained with Pontamine S4B and then observed under a confocal microscope. Representative image of hypocotyl PCW (F). A survey of orthogonal views showing the profile of the hypocotyl PCW (G). Scale bars = 10 μm.

Next, we investigated if the *xxt1 xxt2* phenotype in etiolated hypocotyls was affected by the loss of β-GGM. Plant lines were grown on MS plates in the dark for between 3 and 7 days to measure hypocotyl length. Hypocotyl length differences between the mutants became evident 4 days after germination. Up to day 7, no significant difference was observed between *csla2* and WT seedlings. *xxt1 xxt2* seedlings were shorter than those of WT, consistent with previously published results (Xiao et al., 2016). *csla2 xxt1 xxt2* etiolated seedlings exhibited even shorter hypocotyls than those of *xxt1 xxt2* (Figure 7, A and B). In addition, the *csla2 xxt1 xxt2* seedlings have perturbed growth showing some twisting of hypocotyls (Figure 7A). These results suggest that β-GGM and XyG have connected functions in normal plant development.

### Defects in cell elongation and cellulose microfibril organization

To investigate the developmental changes in β-GGM and XyG mutants, we imaged 4-day-old etiolated seedlings by cryo-SEM and studied the epidermal cell lengths. Compared with WT, the *xxt1 xxt2* mutant exhibited a small reduction in cell length, while the *csla2 xxt1 xxt2* exhibits a larger reduction (Figure 7, C and D). To better visualize the differences in cell expansion along the hypocotyl, we imaged and computationally segmented the hypocotyl cells of the *xxt1 xxt2* and *csla2 xxt1 xxt2* mutants. A heat map of cell length demonstrates that cells are consistently shorter in the *csla2 xxt1 xxt2* mutant along the whole hypocotyl (Figure 7E). The data suggest that cell expansion is further reduced, compared with the loss of XyG alone, by the absence of both β-GGM and XyG.

XyG mutants have revealed the importance of the polysaccharide for normal cellulose fibril arrangements and wall formation (Xiao et al., 2016; Kim et al., 2020). We hypothesized that loss of β-GGM and XyG may affect cell elongation by altering cellulose microfibril arrangements. We processed and stained the cellulose using pontamine fast scarlet 4B dye (Thomas et al., 2017) and imaged epidermal cells using confocal microscopy (Figure 7F). Stained transverse bundles could be observed for WT and *csla2* and these bundles were less defined with areas of missing signal in the *xxt1 xxt2* and *csla2 xxt1 xxt2* mutants, suggesting uneven walls. Orthogonal profiles along the cell length and through the stained walls show thin and even walls for WT and *csla2*, but uneven, “rippled” profiles for *xxt1 xxt2.* This effect is worsened in *csla2 xxt1 xxt2* (Figure 7G). Although the features could be an effect of the processing steps of pontamine staining, they reveal differences in the cellulose arrangements in the cell walls of the mutants that are dependent on the presence of β-GGM and XyG.

### β-GGM has low mobility in PCWs

Hemicellulose polysaccharides that are bound to cellulose are relatively immobile in the cell wall (Bootten et al., 2004). Solid-state NMR (ssNMR) can be used to distinguish more mobile constituents from these relatively immobile polymers. For example, ^13^C cross-polarization (CP)-magic-angle spinning (MAS) ssNMR has been used to study XyG, xylan, and glucomannan bound to cellulose. On the other hand, soluble polymers can be seen by direct polarization (DP)-MAS ssNMR (Metz et al., 1994; Simmons et al., 2016; Cresswell et al., 2021).

Because of the relatively low abundance of β-GGM in plants, and to study a simplified PCW, we exploited Arabidopsis callus cultures of hemicellulose biosynthesis mutants. Compared with seedlings, Arabidopsis callus cultures are relatively homogenous and reproducible between many genotypes. The cells synthesize polysaccharides typical of PCWs (Prime et al., 2000; Nikolovski et al., 2012) and can be labeled by growing with ^13^C-glucose. This enables two-dimensional spectra, in particular using the through-bond refocused INADEQUATE experiment, to be recorded. Such spectra of wild-type callus cells are observed to be complex, and XyG signals dominate (Supplemental Figure S7). Thus, we generated *irx9l xxt1 xxt2* callus cultures to remove both XyG and xylan to simplify the spectra as much as possible, leaving β-GGM as the main hemicellulose. To help in assigning the β-GGM signals in the spectra, we also generated and analyzed a *csla2 xxt1 xxt2* mutant callus, which lacks the β-GGM as well as XyG (Supplemental Figure S6 and Supplemental Table S1).

**Figure 8:**
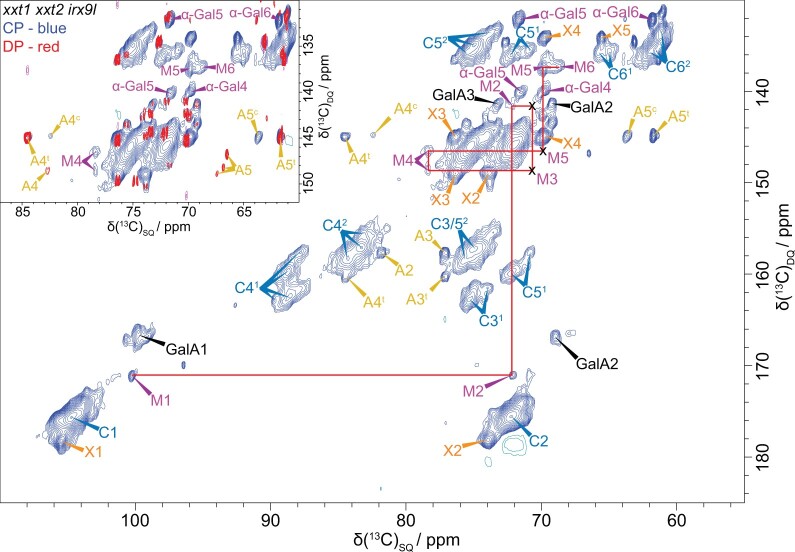
^13^C CP- and DP-refocused INADEQUATE MAS ssNMR spectra show the β-GGM peaks in *irx9l xxt1 xxt2* callus. The β-GGM peaks are labeled: mannose (M) and α-Gal. Also labeled are the main cellulose peaks (domain 1, C^1^; domain 2, C^2^), galacturonic acid (GalA) of pectin, and a terminal xylose (X) linked to an unknown polymer. A terminal arabinose (A^t^) and another arabinose (A^c^) are also labeled. The inset shows an overlay of the CP and DP INADEQUATE spectra for the M5, M6 region. It is clear that M5 and M6 are not visible in the DP spectrum, that is, are not mobile. Spectra were acquired at a ^13^C Larmor frequency of 251.6 MHz and a MAS frequency of 12.5 kHz. The spin-echo duration used was 2.24 ms.

We carried out ssNMR on cell walls without drying or pretreatments, to preserve native arrangements of polymers as much as possible. Figure 8 shows that both Man residues and α-Gal branches of β-GGM can be seen in a ^13^C CP-INADEQUATE MAS NMR spectrum that detects relatively immobile polymers such as cellulose and bound hemicelluloses. The ^13^C NMR chemical shifts of these β-GGM residues are consistent with those from extracted Kiwi fruit glucomannan (Schröder et al., 2001; Supplemental Table S1), supporting the assignments. β-Gal was not detected in the ssNMR spectra, perhaps due to lower abundance or higher mobility of this substitution. The similarity in ^13^C shifts to the previous solution-state assignments suggests that there are no major β-GGM conformational differences in the cell wall. The ^13^C shifts of these β-GGM residues are distinct from those of AcGGM (Terrett et al., 2019; Cresswell et al., 2021), consistent with the different chemical structure of these polymers. Importantly, like XyG and cellulose, β-GGM was not detected in DP-INADEQUATE spectra, in which mobile polymers are seen. Having assigned the β-GGM spectral peaks, we similarly investigated WT cell walls, and confirmed β-GGM is also detectable in CP-INADEQUATE spectra in the presence of the XyG and xylan (Supplemental Figure S7). Due to the low abundance of β-GGM, we have not been able to conduct through-space ssNMR experiments to investigate β-GGM proximity with cellulose. Nevertheless, the experiments indicate that β-GGM has limited mobility in the wall, consistent with binding to cellulose.

## Discussion

XyG has been the focus of eudicot PCW hemicellulose functional studies because of its abundance, and it is the only eudicot hemicellulose with a clear role in cell wall elongation (Burton et al., 2010; Park and Cosgrove, 2015). Here, we report a widespread patterned glucomannan that shows structural and biosynthetic similarities to XyG, which we name β-GGM (Supplemental Figure S1). These two polysaccharides have related roles in cell elongation in plant development, with a role of β-GGM becoming more evident in tissues or mutants without functional XyG. Studies of the role of hemicelluloses in PCW architecture and function should now consider contributions by both polysaccharides.

The glucomannan and XyG biosynthetic enzymes are evolutionarily related (Yin et al., 2009; Wang et al., 2020). The β-GGM backbone is synthesized by CSLA2 in Arabidopsis, a CAZy family GT2 enzyme. Within plant GT2 enzymes, the CSLA enzymes are most closely related to the CSLC family (Mikkelsen et al., 2014; Wang et al., 2020), which are the XyG backbone synthases (Cocuron et al., 2007; Kim et al., 2020). In Arabidopsis, CSLA9 is required for biosynthesis of AcGGM, the random patterned, AcGGM in tissues with SCWs (Goubet et al., 2009). Therefore, there may be functional specialization within the CSLA enzyme family. Whether the ability to make the patterned backbone for β-GGM is intrinsic to specific CSLA enzymes or induced by factors such as MSR proteins (Voiniciuc et al., 2019; Robert et al., 2021) remains to be investigated in plants. The land plant GT2 CSLAs have evolved from the streptophyte algal CSLA/K family (Wang et al., 2020) which is likely to synthesize a mannan. There is no report to our knowledge of glucomannans before the evolution of land plants, so the algal CSLA/K may synthesize a homomannan. The early land plants have been reported to have an AcGGM (Geddes and Wilkie, 1972; Popper and Fry, 2003; Nothnagel and Nothnagel, 2007; Zhang et al., 2014), suggesting that glucomannans are a land plant adaptation. The side chain biosynthesis of β-GGM and XyG is also related. The α-1,6-galactosyltransferase MAGT1 for β-GGM and α-1,6-xylosyltransferase XXTs for XyG are all members of the GT34 family (Scheller and Ulvskov, 2010; Yu et al., 2018). We recently showed that MAGT1 activity has the ability to galactosylate Man in the patterned β-GGM backbone (Yu et al., 2018), but other MAGTs may show preferences for different Man or Glc residue arrangements. Here, we also identified the enzyme making the β-1,2-Gal disaccharide branch, MBGT1. It is from GT47 Clade A, which contains the XyG β-1,2-Gal transferases among many other XyG active enzymes. These extensive similarities in biosynthetic enzymes may imply that β-GGM and XyG have a common ancient evolutionary origin, for example in streptophyte algae where XyG and CSLA/K were present (Mikkelsen et al., 2014, 2021). In this scenario, both polysaccharides persisted through land plant evolution to modern eudicots. Alternatively, the β-GGM biosynthesis may have arisen during land plant evolution from the AcGGM biosynthesis pathway. We have not yet studied the presence of β-GGM across the plant kingdom, and so we are unable to determine yet whether the ability to make β-GGM is ancient or alternatively arose during land plant evolution. Evolution of synthesis β-GGM could require divergence of CSLAs to make the patterned versus unpatterned backbones, specialization of GT34s to add Gal to the patterned backbone, and alteration of a XyG GT47 activity for generation of the β-GGM disaccharide side chains. This second hypothesis would also imply that the β-GGM biosynthesis pathway has evolved to converge on a glucomannan structure closely related to XyG, an idea that raises interesting questions about the importance of this structure for function of both of these polysaccharides.

The molecular structure of hemicellulose polysaccharides influences their solubility and ability to interact with other cell wall components in ways that are not fully understood. It is notable that β-GGM has similarities in structure to XyG, suggesting their backbones and arrangements of branches confer beneficial properties. One distinguishing feature of β-GGM over the previously described AcGGM is the possession of disaccharide branches. What could be the advantage of this structure? The side chains might affect binding to cellulose in the cell wall. In vitro assays showed branches influence the XyG-bacterial cellulose interactions (Lopez et al., 2010), however, there is no clear evidence of an influence on XyG binding in plant cell walls. Second, the side chains may be important for recognition by cell wall modifying enzymes such as XTHs and mannanases (Pena et al., 2004; Schröder et al., 2006; Li et al., 2013; Ishida and Yokoyama, 2022). Third, these side chains might influence solubility of the polymers. The *mur3-1 xlt2* double mutant (with mostly non-substituted XyG composed of XXXG units) can be partially or fully rescued by the addition of d-Gal, l-Ara*f*, or l-Ara*p* at the second or third Xyl*p* residue. This suggests that the disaccharide substitution frequency of XyG is an important parameter for XyG function, but perhaps not the identity or position of the substituted chains (Schultink et al., 2013; Zhu et al., 2018). Thus, a large decrease in XyG substitution in *mur3-3* causes a phenotype, while the smaller decrease in XyG β-Gal in the *xlt2* mutant has no effect. The loss of the side chains may promote inappropriate intracellular interactions of XyG or β-GGM, leading to the formation of membrane aggregates and Golgi secretion disruption (Madson et al., 2003; Zhao et al., 2019). This hypothesis is supported by the fact that the *mur3* phenotype is rescued by plant growth at increased temperature (Shirakawa et al., 1998; Kong et al., 2015). We showed that loss of β-galactosylation of β-GGM exacerbates the *mur3* XyG galactosylation mutant phenotypes, indicating a role for this β-Gal disaccharide side chain.

We also showed using ssNMR that β-GGM is relatively immobile in the cell wall, consistent with binding of this hemicellulose to cellulose. In spruce wood, the AcGGM was found by ssNMR to have close proximity to the cellulose surface. It was further suggested that AcGGM binds to the cellulose surface in a two-fold screw conformation distinct from the soluble AcGGM conformation (Terrett et al., 2019). Here, based on the similarity of ^13^C NMR chemical shifts, we found no evidence for a change in conformation of the β-GGM between solution or in the intact cell wall. Recent molecular dynamics simulations of glucomannan suggest that the backbone Glc residues may promote maintenance of glycosidic bond angles consistent with a two-fold screw, through inter-residue H-bonding as seen in cellulose (Berglund et al., 2016, 2019; Martinez-Abad et al., 2020). Perhaps a consequence of the disaccharide GM repeat is the maintenance of a flattened conformation, unlike that of the flexible conformation AcGGM which has relatively infrequent Glc residues. The simulations also suggested that galactosylation of the Man residue further promotes the formation of two-fold screw ribbon conformation (Berglund et al., 2019). Thus, it is likely that the backbone of β-GGM in solution maintains a flattened conformation that can interact with cellulose without adopting a new shape.

We speculate that β-GGM is likely to interact with cellulose similarly to XyG, but with a few notable differences. XyG, with its glucan backbone, is able to interact with cellulose fibrils. Unlike xylan, which possesses a face that might dock into fibrils and hydrogen bond with the cellulose glucan chains (Busse-Wicher et al., 2016; Simmons et al., 2016; Grantham et al., 2017), XyG is thought to bind to the hydrophobic 100 or 200 cellulose fibril faces through stacking interactions and H-bonding, lying flat with substitutions placed on both sides of the two-fold screw backbone ribbon (Zhao et al., 2014; Benselfelt et al., 2016). Our earlier molecular dynamics simulations suggest β-GGM backbones, which contain alternating Man and Glc, could similarly bind to cellulose (Yu et al., 2018). Since the sugar backbone repeat is GM, in a two-fold screw ribbon each of the Man 2-OH that point out of the hexose ring plane could face away from the cellulose fibril. The β-GGM Glc residues would interact with cellulose as in the XyG backbone. The substitutions could additionally interact with the cellulose surface. However, since substitutions are only present on the alternating residues of Man in β-GGM, these will all lie on one side of the backbone ribbon, unlike XyG where substitutions will lie on both sides of the ribbon. This potentially provides somewhat different hemicellulose–cellulose interaction opportunities.

Studies of Arabidopsis seed mucilage give a hint that β-GGM does functionally interact with cellulose. Although the mucilage β-GGM differs in that it has less β-1,2-Gal at least in part through action of a cell wall β-galactosidase MUM2, the backbone and frequent α-Gal substitution of Man residues are typical of β-GGM. In mucilage, this β-GGM is important for arrangement of the cellulose, because the *csla2* and *magt1* mutants no longer form the normal cellulose rays as in WT (Yu et al., 2014; Voiniciuc et al., 2015). Indications of β-GGM influencing cellulose arrangements also come from staining of the cellulose in etiolated hypocotyls, since altered arrangements were seen in the mutants lacking both β-GGM and XyG.

The structural similarity of β-GGM and XyG led us to hypothesize that they may play connected functions in the cell wall during growth and development. Previously, our knowledge of glucomannan function from Arabidopsis molecular genetics indicated a role mainly limited to seed mucilage and in embryogenesis (Goubet et al., 2003, 2009; Yu et al., 2014, 2018; Voiniciuc et al., 2015; Somssich et al., 2021). Our results support the idea that XyG conceals the importance of β-GGM in many tissues. For example, the Arabidopsis *csla2* β-GGM mutant shows few phenotypes in the plant, but it does have altered adherent mucilage (Yu et al., 2018). Notably, in the mucilage XyG is undetectable (Haughn and Western, 2012). Studies of the β-GGM and XyG backbone synthesis mutants also support a connection in function. The *csla2 xxt1 xxt2* mutant had more severe growth phenotypes than the *xxt1 xxt2* alone, again showing the role of β-GGM is partly obscured by XyG. We also showed that the loss of the β-GGM disaccharide side chain exacerbated the severity of XyG galactosylation mutant phenotypes, even though phenotypes were not observed in the presence of normal XyG. β-GGM and XyG are therefore connected in their functions, and they are both involved in cell expansion in various tissues. The relatively mild phenotypes of XyG mutants and β-GGM mutants are in part due to a level of functional redundancy of these hemicelluloses. It might be that loss of yet further hemicelluloses, including xylan, will reveal more severe impacts on wall function. The implication of our results is also that studies of XyG function have been hindered by the presence of β-GGM. β-GGM now needs to be studied alongside XyG in studies of hemicellulose function in plant cell expansion and development.

## Materials and methods

### Plant materials

Arabidopsis (*A. thaliana*) plants used in this work were from Col-0 ecotype. The various mutants are: *mbgt1-1* (SALK_065561), *mbgt1-2* (SAIL_852_F05C), *csla2* (SALK_065083), *csla9* (SALK_071916), *magt1* (SALK_061576), *xxt1* (SAIL_785_E02), *xxt2* (SALK_101308), *mur3-1* (Reiter et al., 1997), *mur3-3* (*SALK_141953*), *xlt2* (GABI_552C10), *fut1* (*mur2-1*) (Reiter et al., 1997), *irx9l* (SALK_037323), *mum2-10* (SALK_011436), *csla2 clsa9* (Goubet et al., 2009), and *xxt1 xxt2* (Cavalier et al., 2008). The *csla2 xxt1 xxt2* triple mutant was generated by crossing *csla2* and *xxt1 xxt2*, the *irx9l xxt1 xxt2* triple mutant was generated by crossing *irx9l* and *xxt1 xxt2*, the *mbgt1-1 mur3-1* double mutant was generated by crossing *mbgt1-1* and *mur3-1*, and the *mbgt1-1 mur3-3* double mutant was generated by crossing *mbgt1-1* and *mur3-3*. The homozygous lines were identified by PCR. The primers used for genotyping are shown in Supplemental Table S3.

### Plant growth conditions

Plants were grown in controlled-environment chambers. Arabidopsis seeds were surface sterilized, sown on half Murashige and Skoog (MS) medium with 1% sucrose, stratified in darkness for 48 h at 4°C, and then germinated at 21°C under white light (MASTER TL-D Super 80 58W/840 1SL/25 [Philips] and Sylvania 58W T8 5ft Grolux Tube [Sylvania]; 150 mmol m^−2^ s^−1^) with a 16-h light/8-h dark cycle. After 10 days, the seedlings were transferred to soil (Advance M2, ICL Levington) and grown in growth chambers under the same conditions. Arabidopsis liquid callus cultures were generated from roots and maintained as described in Prime et al. (2000). Uniformly labeled ^13^C glucose (2%; Cambridge Isotope Laboratories, CLM-1396) was used as a carbon source in order to grow ^13^C enriched callus for ssNMR analysis.

Rosette leaves were harvested at 6 weeks, young stems at 30 days, siliques at 6 weeks, and mature stems at 8 weeks. The plant height, the number of rosette branches, and the number of cauline branches were measured at 7 and 8 weeks. A rosette branch was defined as one originating from axils on the unexpanded stem, while the cauline branch was defined as one originated from the expanded segment of the inflorescence stem (Keller et al., 2006). All experiments were performed on at least three independently harvested sets of plant material.

*Nicotiana benthamiana* plants were grown at 21°C under 16-h light/8-h dark conditions. Leaves of 4-week-old *N. benthamiana* were used for infiltration.

### Hypocotyl and cell measurements

Seeds were surface-sterilized, sown on MS plates, and stored at 4°C for 3 days. Seeds were exposed to light for 6 h to stimulate germination, then wrapped in two layers of aluminum foil and grown for 2–7 days at 21°C. Plates with etiolated seedlings were scanned using an HP Scanjet 8300 scanner at 600 dpi, and hypocotyl length was measured using ImageJ. To measure cell length, 4-day-old etiolated seedlings were firstly analyzed with cryo-SEM. Four-day-old etiolated seedlings were mounted onto carbon pad stubs, frozen, and then coated with platinum and maintained at −145°C as described previously (Lyczakowski et al., 2019). Images were acquired on a Zeiss EVO HD15 using a backscattered electron detector and an accelerating voltage of 25 kV with a working distance of >15 mm. Cell length measurements were taken for cells at the base of the hypocotyl using ImageJ software.

For generating the heat maps comparing cell length between *xxt1 xxt2* and *csla2 xxt1 xxt2* mutants, 4-day-old etiolated seedlings were submersed in 0.1 mg mL^−1^ propidium iodide for 3 min, washed briefly in water, and then mounted in water on a microscope slide with a coverslip. The slide was mounted on an inverted Leica DMi8 SP8 confocal microscope fitted with a 10× objective lens. Whole seedlings were imaged for fluorescence in 3D using the tile scan feature of the Leica LAS X Navigator software module and the tiles fused to generate a single *z*-stack file covering the whole hypocotyl region. The files were converted to tiff stacks and imported into MorphoGraphX (de Reuille et al., 2015). Voxels were averaged (XRad, YRad, ZRad = 2) and the following software tools implemented in this order: Edge detect (20,000), fill holes, closing, Marching cubes surface, located and deleted erroneous volumes manually, smooth mesh, subdivide, smooth mesh, project signal (5–10), Gaussian blur (2 px radius), draw seeds as long lines down the center of each cell, watershed segmentation, corrected incorrect segmentations by drawing new seeds, and resegmenting. An updated version of MorphoGraphX was obtained from Richard Smith (John Innes Centre, Norwich) which allows heat maps to be generated based on major axis length. These length heat maps were scaled from 0 to 200 micron range.

### Cellulose fluorescent staining and imaging

Four-day-old seedlings were stained according to the protocol described previously (Landrein et al., 2013). In our hands, cells in the upper portion of the hypocotyl stained uniformly while cells in the lower half did not. Expanded cells below the apical hook were therefore selected for imaging using an upright Leica SP8 confocal microscope fitted with a 552-nm laser for excitation and 63× 1.4 NA oil immersion lens for imaging. Confocal optical sections were taken that covered the full depth of staining. Representative images in Figure 5 are taken from the middle of the upper cell wall surface with two consecutive sections averaged to aid observations of cellulose patterns. Orthogonal views were created by drawing line regions of interest along the length of the center of cells using ImageJ and then using the reslice option.

### Preparation of soluble hemicelluloses

Dry and clean seeds were shaken in dH_2_O in a tube without beads for 30 min at 30 Hz in a Retsch MM400 mill. The seed suspension was centrifuged at 100 × *g* for 1 min. The supernatant was harvested and the seeds were washed twice with dH_2_O by repeating the centrifugation to get naked seeds. The mucilage supernatants were collected and used for mucilage analysis. Callus was harvested and washed with ddH_2_O to remove medium. Alcohol insoluble residue (AIR) from stems, leaves, seed mucilage, naked seeds, siliques, callus, and etiolated seedlings was prepared as previously described (Goubet et al., 2009; Yu et al., 2018). Thirty milligrams of AIR was treated with 2.5 mL of 4 M NaOH at room temperature (RT) for 1 h and centrifuged at 4000 rpm for 15 min. In order to neutralize the NaOH, prior to enzymatic digestion, the supernatants were loaded onto a PD-10 desalting column (GE Life-Science) and eluted with 50 mM ammonium acetate (pH 6.0) according to the manufacture instruction. The eluent contained the majority of the de-acetylated hemicelluloses and was aliquoted into tubes for 25 mannan digestion reactions or 50 XyG digestion reactions.

### Enzymatic digestions of extracted hemicelluloses

For mannan analysis, the hemicelluloses eluted from PD-10 were digested with an excess of *Cellvibrio japonicus* Man26A (*Cj*Man26A) mannanase (1 μL of 3.8 mg/mL; University of Newcastle) or *Aspergillus nidulans* GH5 (*An*GH5) mannanase (1 μL of 3.5 mg/mL; Novozymes) in 50 mM ammonium acetate (pH = 6.0) at 37°C overnight. Mannanases were de-activated after digestion with a heat treatment at 105°C for 10 min. Mannanase products were then digested overnight with *Aspergillus niger* GH35 β-galactosidase (1 μL of 6 mg/mL; Megazyme) or *Cellvibrio mixtus* GH27 α-galactosidase (1 μL of 1 mg/mL; Prozomix) in 50 mM ammonium acetate (pH 6.0) at 37°C to remove the β-Gal or α-Gal side chains. For sequential digestion, enzymes used were: *A. niger* GH3 β-glucosidase (1 μL of 2 mg/mL; Novozymes) and *C. mixtus* GH5 β-mannosidase (1 μL of 0.8 mg/mL; University of Newcastle). The digestion conditions were 50 mM ammonium acetate (pH 6.0) at 37°C for 4 h with excess enzymes to complete digestion. After each reaction, samples were boiled at 100°C for 10 min to denature the enzyme. Samples were then dried at 60°C in vacuo.

For XyG analysis, the eluted hemicellulose fractions were digested with an excess of *Paenibacillus pabuli* XG5 (*Pp*XG5) xyloglucanase (1 μL of 0.6 mg/mL; Novozymes) in 50 mM ammonium acetate (pH 6.0) at 37°C for 18 h.

For xylan analysis, the eluted hemicellulose fractions were digested with an excess of *Neocallimastix patriciarum* GH11 (*Np*GH11) xylanase (1 μL of 16 mg/mL; Megazyme) as previously described (Mortimer et al., 2010).

### Oligosaccharide fingerprint analysis by PACE

Samples and a standard mixture of 5 nmol each of mannose and mannnooligosaccharides with DP 2–6 (Megazyme) were derivatized with 8-aminonaphthalene-1,3,6-tresulfonic acid (Invitrogen) as described previously (Goubet et al., 2002). After drying, the samples were re-suspended in 100 μL of 3 M urea, of which 2 μL was loaded onto the PACE gels. The samples were run and visualized using a G-box equipped with a trans-illuminator with long-wavelength light tubes (365 nm) and a short pass filter (500–600 nm) as described previously (Goubet et al., 2002). All analyses of oligosaccharides were repeated a minimum of three times.

### Preparation of oligosaccharides for MS

Following enzymatic digestion, released peptides and enzymes were removed using reverse-phase Sep-Pak C18 cartridges (Waters) as previously described. The oligosaccharides were reductively aminated with 2-aminobenzamide (2-AB), using optimized labeling conditions. The labeled samples were then purified from reductive amination reagents using a GlycoClean S cartridge (Prozyme) as described previously (Tryfona et al., 2012).

### Hydrophilic interaction liquid chromatography-MALDI-ToF MS/MS

Capillary hydrophilic interaction liquid chromatography was carried out using an LC-Packings Ultimate system (Dionex), using optimized elution conditions and robot harvest systems. After air drying, the sample spots were overlaid with 2,5-dihydroxybenzoic acid matrix and analyzed by MALDI-ToF/ToF-MS/MS as described previously (Tryfona et al., 2012).

### Separation of oligosaccharides by SEC

Arabidopsis young stem AIR (500 mg), hydrolyzed with an excess of enzymes (first *Cj*Man26A and then by a combination of β-glucosidase, β-mannosidase, and α-galactosidase), was prepared as described above, and lyophilized. Samples were re-suspended in 2 mL dH_2_O, loaded onto a gravity-driven preparative Bio-Gel P2 column (190 × 2.5 cm; Bio-Rad), equilibrated, and run in 20 mM ammonium acetate pH 6.0. Fractions were collected and dried in vacuo. Fractions of interest were studied by PACE.

### Solution-state NMR

Following SEC, lyophilized samples were re-suspended in D_2_O (700 µL; 99.9% purity) and transferred to a 5-mm NMR tube. NMR spectra were recorded at 298 K with a Bruker AVANCE III spectrometer operating at 600 MHz equipped with a TCI CryoProbe. ^1^H chemical-shift assignments were primarily obtained using ^1^H–^1^H total correlation spectroscopy (TOCSY) and rotating frame Overhauser effect spectroscopy (ROESY). The H-1/H-2 peaks in a double quantum (DQ) filtered correlation spectroscopy (DQFCOSY) were used to remove ambiguities in the assignments of H-2. ^13^C assignments were obtained using ^13^C HSQC and H2BC experiments (although the latter was incomplete due to the low concentration of the sample) (Cavanagh et al., 1995; Nyberg et al., 2005); the mixing times were 70 and 200 ms for the TOCSY and ROESY experiments, respectively. Chemical shifts were measured relative to internal acetone (δ(^1^H) = 2.225, δ(^13^C) = 31.07 ppm). Data were processed using the Azara suite of programs and chemical-shift assignment was performed using CCPN Analysis v2.4 (Vranken et al., 2005).

### Protein expression and immunoblot analysis

3×Myc tagged *MBGT* (At4g13990) coding sequence was PCR amplified from synthetic DNA (IDT) using primers described in Supplemental Table S3. The PCR product was cloned under a 35S promoter in a pEAQ vector (Sainsbury et al., 2009) using *Nur*I site. PgGUX was prepared as previously described (Lyczakowski et al., 2017). Infiltration of *N. benthamiana*, microsome isolation, and immunoblot analysis of membrane preparations were all performed as previously described (Lyczakowski et al., 2017). For the immunoblot analysis, a rabbit anti-c-Myc IgG (polyclonal, 1:2,000; Santa-Cruz Biotechnology, A14) and a goat anti-rabbit IgG HPR conjugate (1:10,000; Bio-Rad, 170-6515) were used as a primary and secondary antibody, respectively.

### β-Galactosyltransferase activity assay

Adherent mucilage hemicelluloses from WT seeds, rich in β-GGM lacking β-Gal, were prepared as previously described (Yu et al., 2018). *mbgt-1* soluble hemicellulose from young stems was prepared as above. WT adherent mucilage hemicelluloses and *mbgt-1* young stem hemicelluloses aliquots were dried and used as acceptors for in vitro β-Gal transfer reaction. UDP-Gal (5 mM) was replaced with water in certain reactions to control for endogenous activity. Reaction was performed for 5 h at RT and was terminated by heating the samples at 100°C for 10 min. The polysaccharides were extracted using methanol and chloroform as previously described (Lyczakowski et al., 2017). Extracted polysaccharides were digested with *Cj*Man26A and analyzed with PACE.

### Phylogeny

The bulk of the GT47 Clade A sequences were downloaded as an orthologous cluster from the comparative genomics platform Plaza Dicots 4.5, Plaza Monocots 4.5 (Van Bel et al., 2018), and Plaza Gymnosperms 3.0 (Proost et al., 2015), but were supplemented with the results of HMMER (http://hmmer.org/) and TBLASTN (Altschul et al., 1990, 1997) searches of additional published genomes (Hori et al., 2014; Filiault et al., 2018; Li et al., 2018; Weston et al., 2018; Chen et al., 2019; Zhang et al., 2020) using an Arabidopsis GT47 Clade A HMM or the *At*MUR3 protein sequence as a query, respectively. For the GT47-A tree, sequences were aligned with MAFFT (Katoh et al., 2002; Katoh and Standley, 2013) and truncated to their predicted GT47 domain (corresponding to residues 156–539 of *At*MUR3) using a custom Python script (https://www.python.org/). Substantially truncated and very poorly aligned sequences were removed from the alignment manually. Prottest3 (Darriba et al., 2011) was used to determine an appropriate substitution model (LG), and the tree was built with FastTreeMP (Price et al., 2010) with 100 bootstraps. The alignments used to generate the phylogeny are provided as Supplemental Files S1 and S2.

### Preparation of callus sample for ssNMR

^13^C labeled callus was harvested and washed six times with unlabeled callus medium to remove the ^13^C glucose. Then the callus was frozen in liquid N_2_ and stored at −80°C overnight. Frozen callus was ground into powder in liquid N_2_, thawed on ice, and centrifuged at 15,000 rpm at 4°C, removing excess liquid, twice to obtain moist callus sample for ssNMR.

### Solid-state NMR

Solid-state MAS NMR experiments were performed using Bruker (Karlsruhe, Germany) AVANCE NEO ssNMR spectrometers, operating at ^1^H and ^13^C Larmor frequencies of 1000.4 MHz and 251.6 MHz and 850.2 and 213.8 MHz, respectively, with 3.2 mm double-resonance E^free^ MAS probes. Experiments were conducted at an indicated temperature of 283 K at an MAS frequency of 12.5 kHz on both spectrometers. The ^13^C chemical shift was determined using the carbonyl peak at 177.8 ppm of l-alanine as an external reference with respect to tetramethylsilane. Both ^1^H−^13^C CP, with ramped (70%–100%) ^1^H rf amplitude and 1 ms contact time, and DP were used to obtain the initial transverse magnetization (Metz et al., 1994). While CP emphasizes the more rigid material a short, 2 s, recycle delay DP experiment was used to preferentially detect the mobile components. Two-dimensional DQ correlation spectra were recorded using the refocused INADEQUATE pulse sequence which relies upon the use of isotropic, scalar J coupling to obtain through-bond information regarding directly coupled nuclei (Lesage et al., 1997, 1999; Fayon et al., 2005). The carbon 90° and 180° pulse lengths were 3.5–4.3 and 7.0–8.6 μs, respectively, with 2τ spin-echo evolution times for a (π–τ–π/2) spin-echo of 4.48 ms. SPINAL-64 ^1^H decoupling was applied during both the evolution and signal acquisition periods at a ^1^H nutation frequency of 70–80 kHz (Fung et al., 2000). The acquisition time in the indirect dimension (*t*_1_) was 5.0–6.0 ms for the CP-INADEQUATE and 5.5 ms for the DP INADEQUATE experiment. The spectral width in the indirect dimension was 50 kHz for both with 192–416 acquisitions per *t*_1_ FID for the CP-INADEQUATE and 80 acquisitions for the DP INADEQUATE experiments. The States-TPPI method was used to achieve sign discrimination in *F*_1_. The recycle delay was 2 s for both CP INADEQUATE and DP INADEQUATE experiments. The spectra were obtained by Fourier transformation into 4 K (*F*_2_)× 2K (*F*_1_) points with exponential line broadening in *F*_2_ of 50 Hz for CP and 20 Hz for DP experiments, respectively, and squared sine bell processing in *F*_1_. All spectra obtained were processed and analyzed using Bruker Topspin version 3.6.2.

## Accession numbers

Sequence data from this article can be found in the Arabidopsis Genome Initiative or GenBank/EMBL databases under the following accession numbers: At4g13990 (MBGT1), At5g22740 (CSLA2), At5g03760 (CSLA9), At2g22900 (MAGT1), At3g62720 (XXT1), At4g02500 (XXT2), At1g27600 (IRX9L), At2g20370 (MUR3), At5g62220 (XLT2), At2g03220 (FUT1), and MUM2 (At5g63800).

## Supplemental data

The following materials are available in the online version of this article.

**Supplemental Figure S1.** Schematic structures of PCW hemicellulose.

**Supplemental Figure S2.** PACE gels of control samples of un-digested material and enzymes.

**Supplemental Figure S3.** Structural analysis of α-galactosylated mannan oligosaccharides from *csla9* young stem.

**Supplemental Figure S4.** Patterned β-GGM is widely present in eudicots.

**Supplemental Figure S5.** Loss of XyG does not affect the production of CSLA2 β-GGM, or vice versa.

**Supplemental Figure S6.** Arabidopsis callus hemicelluloses analyzed by PACE.

**Supplemental Figure S7.** ssNMR of WT Arabidopsis callus.

**Supplemental Table S1.** Chemical shifts (in ppm) for different moieties obtained with solution or solid state NMR.

**Supplemental Table S2.** Plant species used for the phylogenetic tree.

**Supplemental Table S3.** Primers used in this study.

**Supplemental File S1.** Sequence alignment of amino acid sequences of GT47A for phylogeny.

**Supplemental File S2.** Phylogenetic tree of GT47A.

## Supplementary Material

koac238_Supplementary_Data

## Notes

L.Y. and P.D. conceived and designed the study. L.Y. conducted most of the experiments and analyzed the data. X.Y. made callus for ssNMR. R.C., R.D., and S.P.B. conducted the ssNMR experiments. ssNMR data were analyzed by R.C., R.D., Y.Y., L.Y., and P.D. J.J.L. made the constructs and contributed to the MBGT protein expression for in vitro assay. R.W. performed the microscopy. L.F.L.W. did the phylogenetic analysis and statistics. Y.Y. measured the plant growth phenotypes. Y.Y. and K.I. performed some of the PACE analysis. X.Y. and J.W.-R. performed the crosses of plants. K.S. performed the solution NMR and analyzed the data. K.B.R.M.K. assisted with guidance on the use of glycoside hydrolase enzymes and S.C. tested the enzyme specificities. O.M.T. and H.T. contributed to the data interpretation and project discussion. L.Y., Y.Y., and P.D. wrote the manuscript. J.J.L., L.F.L.W., Y.Y., O.M.T., and K.I. assisted writing the manuscript. All authors commented on and approved the final manuscript.

The author responsible for distribution of materials integral to the findings presented in this article in accordance with the policy described in the Instructions for Authors (https://academic.oup.com/plcell) is: Paul Dupree (pd101@cam.ac.uk).
