# Supplementary material for: Eudicot primary cell wall glucomannan is related in synthesis, structure, and function to xyloglucan
Source: Plant Cell. 2022 Aug 4;34(11):4600–22. doi: 10.1093/plcell/koac238 (PMC9614514; doi:10.1093/plcell/koac238)
Supplement: koac238_Supplementary_Data [file koac238_supplementary_data.zip › koac238_Supplementary_Data/Yu2022Supplemental Data.pdf]

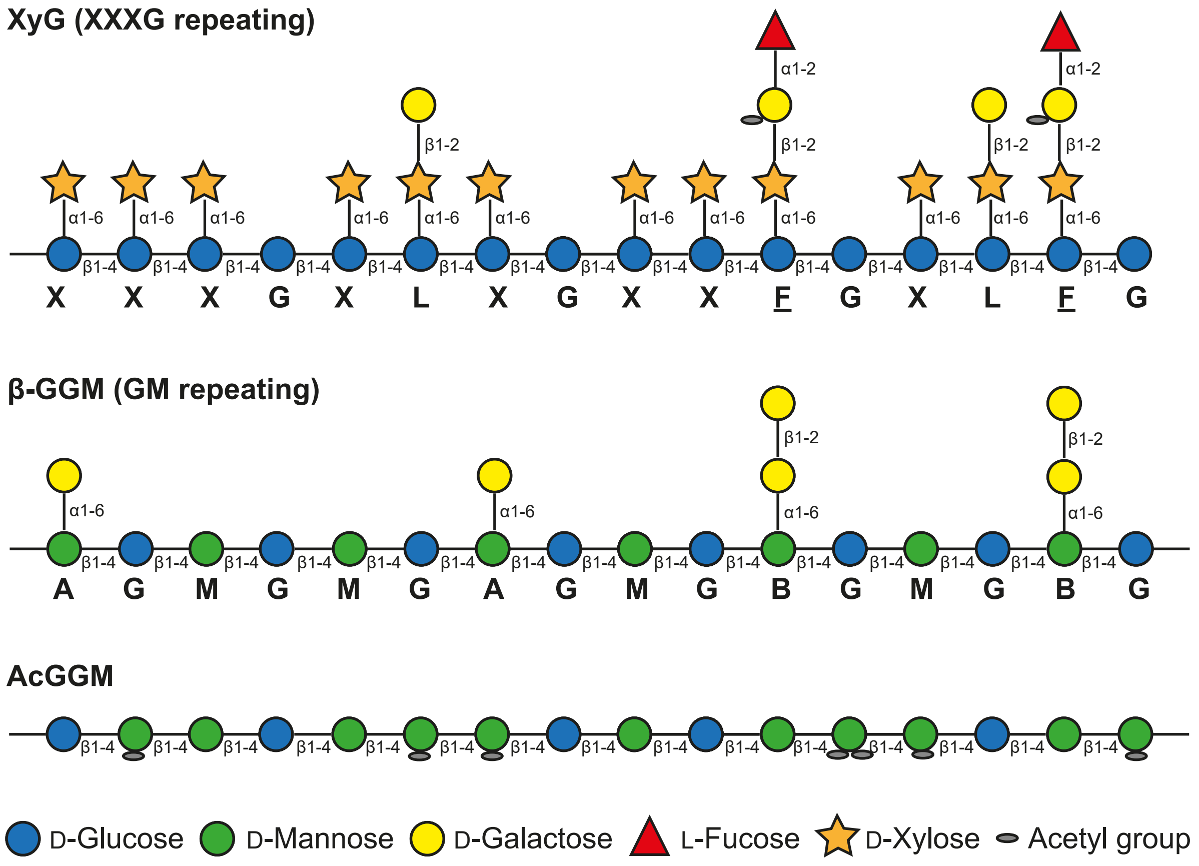

Supplemental Figure S1. Schematic structures of primary cell wall hemicellulose. This supports Figure 1, 2, and 3 to understand two distinctive glucomannan structures as well as the structural similarity of β-GGM with xyloglucan.

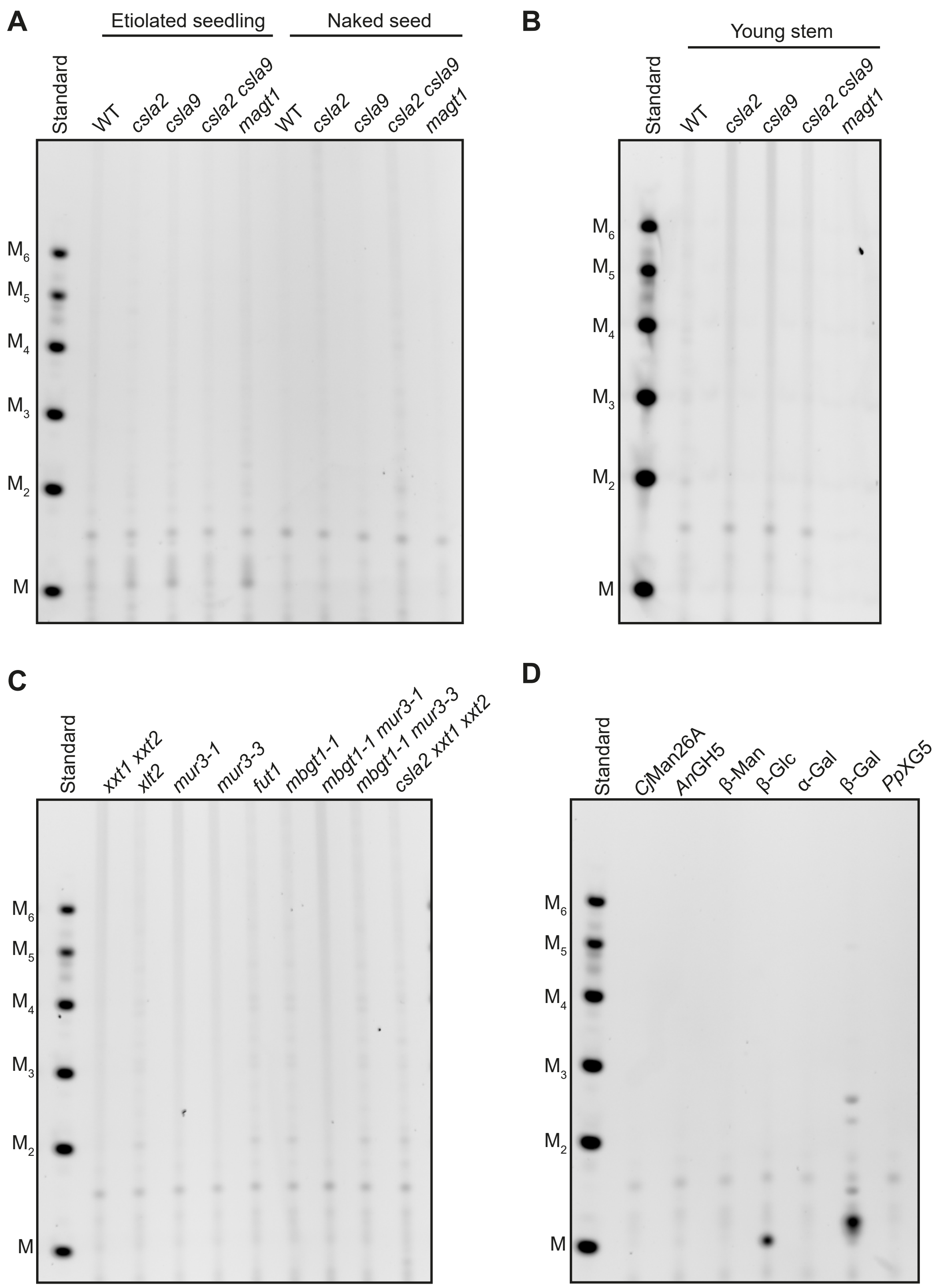

Supplemental Figure S2. PACE gels of control samples of un-digested material and enzymes. A, un-digested control of etiolated seedling and naked seed samples. B, un-digested control of young stem samples. C, un-digested control of young stem samples of XyG related mutants. D, Background bands brought by enzymes used in this work, supporting that all PACE results were not contamination by enzymes themselves. Markers M to M6 are shown. Markers M to M_6 are shown. This supports all PACE results in Figure 1, 2, 3, and Supplemental Figure S3-S6.

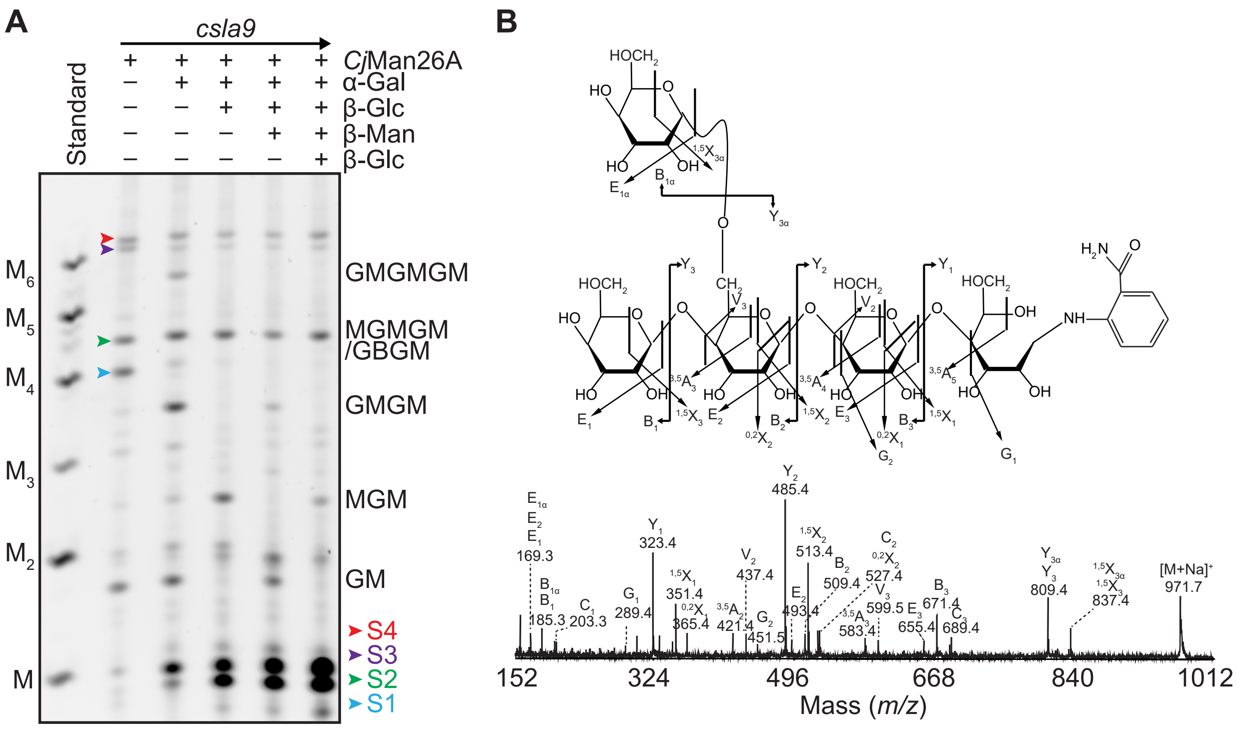

Supplemental Figure S3. Structural analysis of α-galactosylated mannan oligosaccharides from *csla9* young stem. A, Analysis of α-galactosylated mannan by PACE. *csla9* young stem was digested with *Cj*Man26A first and the resultant oligosaccharides were digested sequentially with α-galactosidase (α-Gal), β-glucosidase (β-Glc), and β-mannosidase (β-Man) enzymes. α-Galactosylated mannan oligosaccharides have Glc-Man repeating units. Markers M to M_6 are shown. B, Hex5 (S1) in Figure 2B was analysed by high-energy CID MS/MS. The first Man at the non-reducing end is decorated with a single α-1,6-Gal. This supports Figure 2.

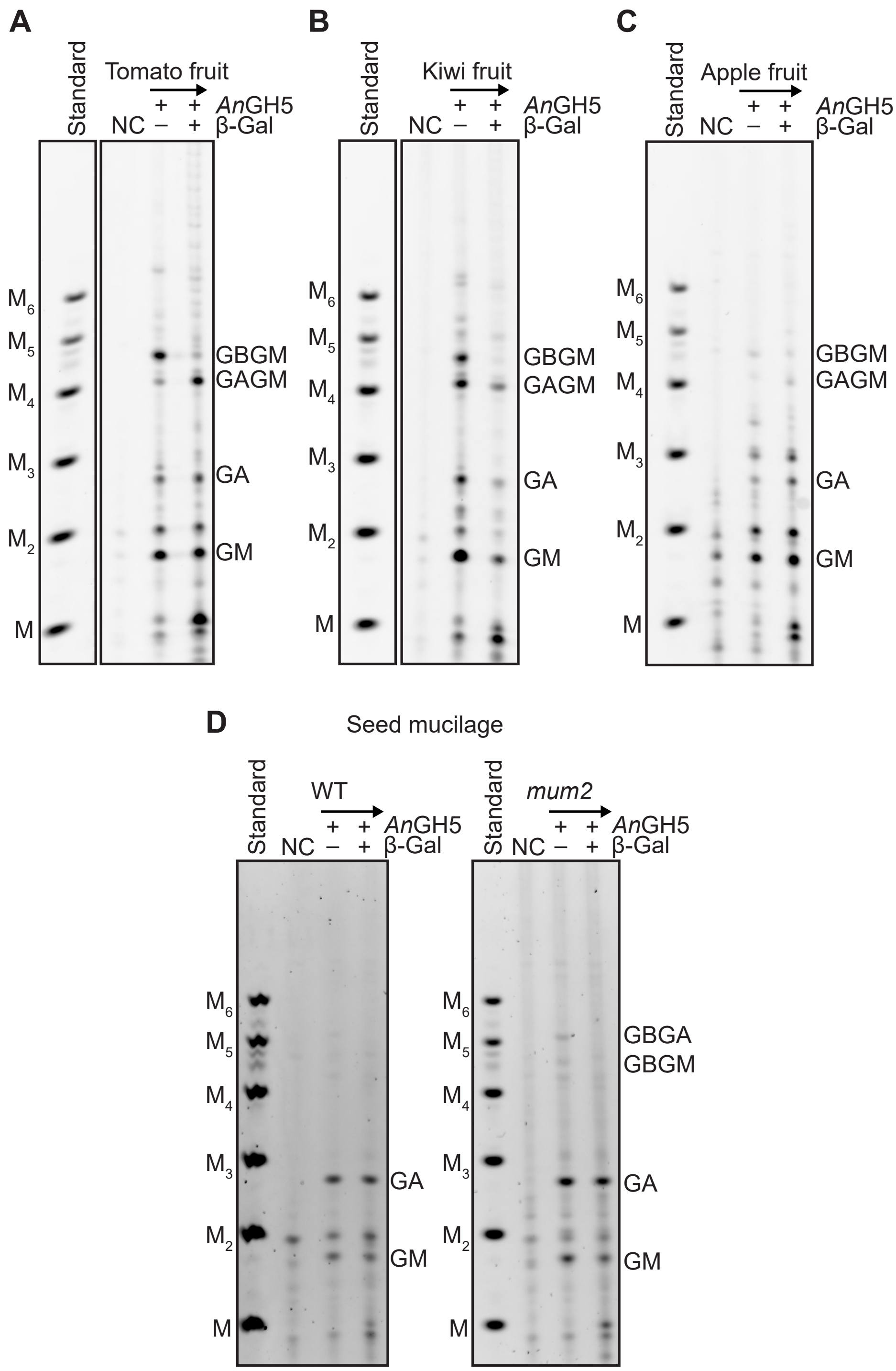

Supplemental Figure S4. Patterned β-GGM is widely present in eudicots. A to C, Mannan from tomato fruit and kiwi fruit was analysed. *An*GH5 products of AIR were digested with β-galactosidase (β-Gal) to test the presence of β-GGM. NC indicates a control without enzyme. D, Arabidopsis seed mucilage from WT and *mum2* was digested with *An*GH5. β-GGM was detected in *mum2* mucilage. M, Man; G, Glc; Markers M to M_6 are shown. This supports Figure 1.

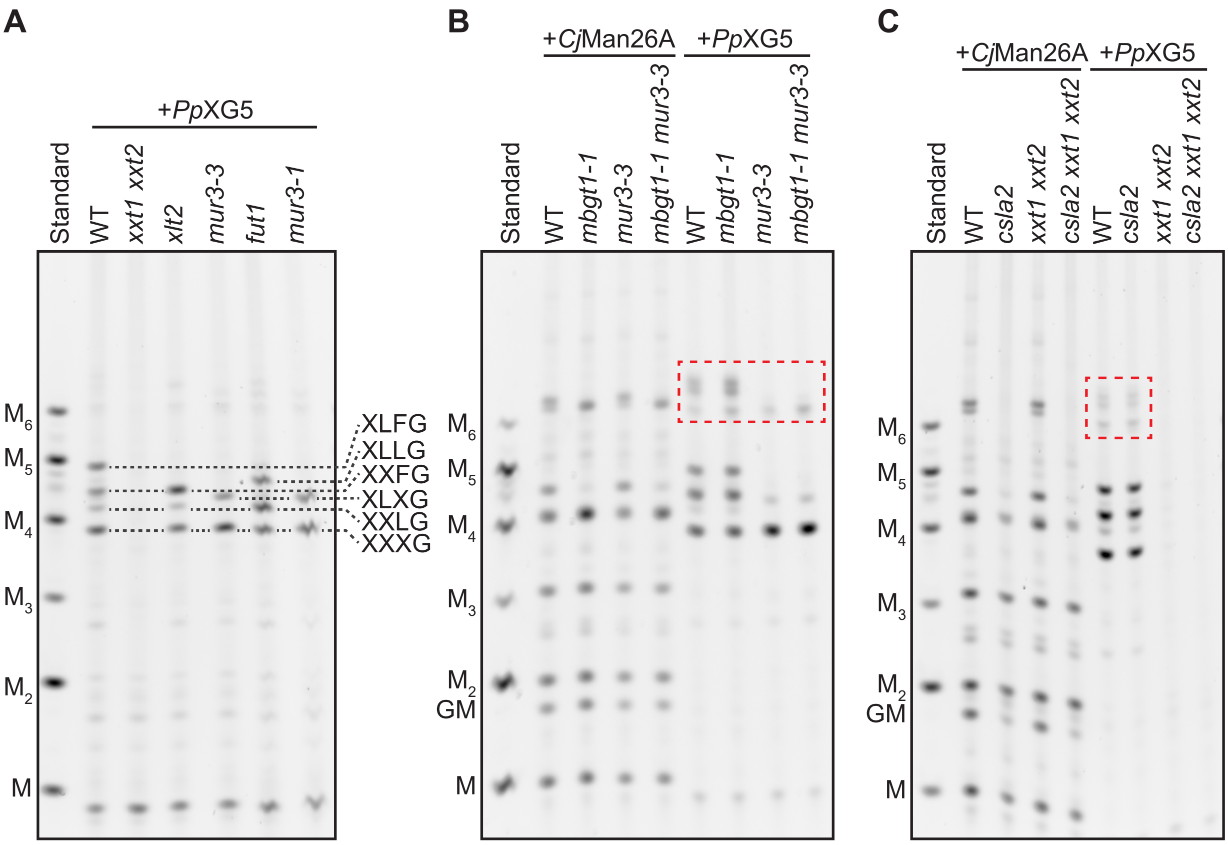

Supplemental Figure S5. Loss of XyG does not affect the production of CSLA2 β-GGM, or vice versa. Five-week-old young stem was used for the following digestion. A, Assignment of *Pp*XG5 products of XyG digestion with PACE. The assignment is enabled by XyG-related mutants. B, Structure of XyG and β-GGM in *mur3-3* or *mbgt1-1* mutants. C, Structure of XyG and β-GGM in *xxt1 xxt2* and *csla2* mutants. Bands in red dashed lines are XyG oligosaccharide dimers. M: Man; G: Glc. Markers M to M_6 are shown. This supports Figure 5, 6, and 7 to confirm the hemicellulose structures in the mutants.

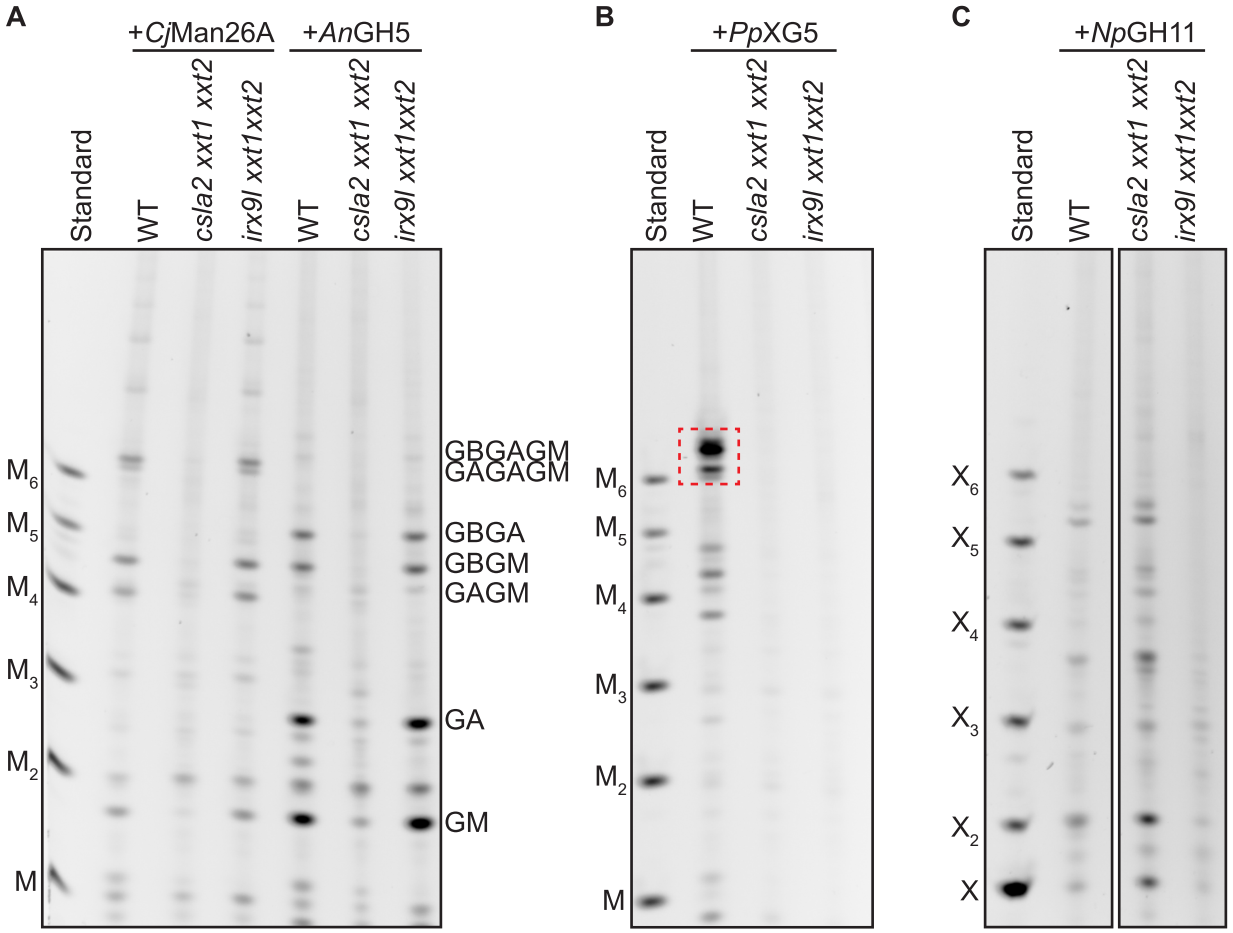

Supplemental Figure S6. Arabidopsis callus hemicelluloses analysed by PACE. A, Callus mannan was analysed by both *Cj*Man26A and *An*GH5 mannanases. *csla2*-related mutants lack mannan accessed by *Cj*Man26A and *An*GH5. B, Callus XyG was analysed by *Pp*XG5. Bands in red dashed lines are XyG oligosaccharide dimers. C, Callus xylan was analysed by *Np*GH11. *irx9l*-related mutants lack xylan accessed by *Np*GH11. M: Man; X: Xyl. Markers M to M_6 and X to X_6 are shown. This supports Figure 8 to confirm the hemicellulose structures in the mutants.

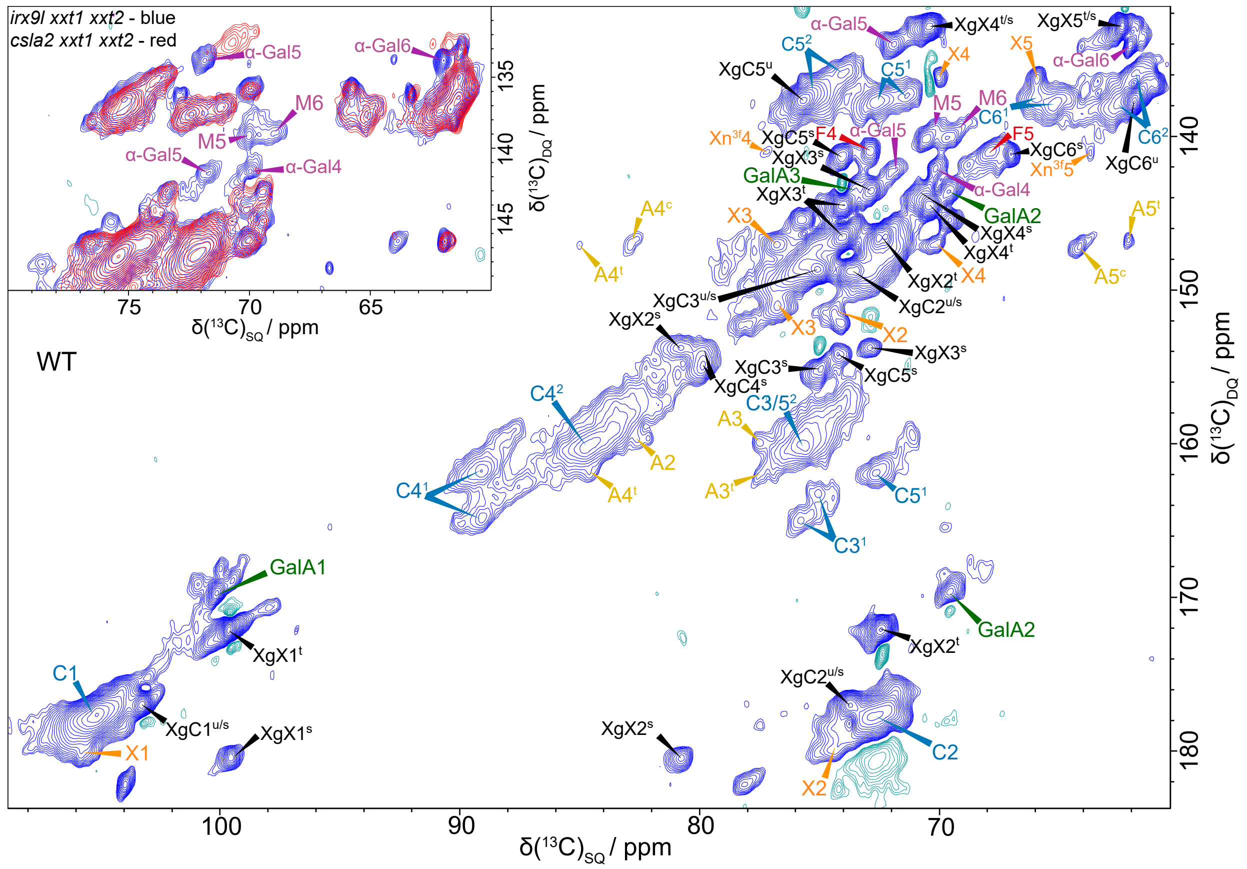

Supplemental Figure S7. Solid-state NMR of WT Arabidopsis callus. The carbohydrate region of a refocussed CP-INADEQUATE ^{13}C MAS NMR spectrum of ^{13}C enriched WT callus with the β-GGM Man peaks M5 and M6 labelled. Xyloglucan is also labelled as well as carbons in the major polysaccharides: galacturonic acid (GalA), terminal Xyl (X) and α-Gal and two arabinoses (A^t and A^c). The terminal arabinose is labelled t and the other arabinose c. For cellulose, the environments have been split into two groups, domain 1 and 2 cellulose (C^1 and C^2). For xyloglucan, 5 sets of environments are seen depending on the substitution, labelled as unsubstituted backbone Glc (XyC^u), substituted backbone Glc (XyC^s), terminal Xyl on XyG (XgX^t), substituted Xyl (XgX^s), and Fuc (F). Assignments are listed in Supplemental Table 1. The inset shows an overlay for the M5, M6 region of a CP INADEQUATE spectrum of ^{13}C enriched *irx9l xxt1 xxt2* callus (blue) with that of *csla2 xxt1 xxt2* callus. As expected, the β-GGM (M and α-Gal) peaks are missing from the *csla2 xxt1 xxt2* spectrum. Spectra were acquired at a ^{13}C Larmor frequency of 213.8 MHz for WT callus and *csla2 xxt1 xxt2* and 251.6 MHz for *irx9l xxt1 xxt2*. The MAS frequency was 12.5 kHz and the spin-echo duration was 2.24 ms. This supports Figure 8.

Supplemental Table S1. Chemical shifts (in ppm) for different moieties obtained with solution or solid state NMR

Solution NMR------ ^{1}H and ^{13}C NMR chemical shifts for the GGM oligosaccharide

Name	Type, moiety	H1/C1	H2/C2	H3/C3	H4/C4	H5/C5	H6/C6	References
β-GGM	Substituted α-Gal	5.2/99.3	3.94/78.5	4.16/74.3	3.76/70.8	3.5	n/a	This work
β-GGM	β-Gal	4.57/105.5	3.62/71.4	3.65/73.3	3.73/71.4	n/a	n/a	This work

Solid State NMR------ ^{13}C NMR chemical shift of polysaccharides in WT callus

Name	Type, moiety	C1	C2	C3	C4	C5	C6	References
Spruce AcGGM	Man	101.9	72.0	n/a	80.4	75.8	61.6	Terrett et al., 2019
Spruce AcGGM	Acetylated Man	100.9	71.9	75.9	80.4	75.8	61.6	Terrett et al., 2019
Kiwi fruit GGM	Substituted Man	100.4	70.9	70.4	78.1	69.6	68.6	Schroder et al., 2001
β-GGM	Man	100.4	72.4	71.0	78.6	70.1	68.9	This work
Kiwi fruit GGM	Un-substituted α-Gal	99.2	71.9	71.5	76.9	69.6	60.4	Schroder et al., 2001
Kiwi fruit GGM	Substituted α-Gal	98.9	78.1	70.4	69.6	75.5	61.3	Schroder et al., 2001
β-GGM	α-Gal	n/a	n/a	n/a	70.1	71.9	62.0	This work
Xyloglucan	Terminal α-Xyl	99.5	72.4	74.0	70.2	62.0	-	York et al., 1993
Xyloglucan	Terminal α-Xyl	99.7	72.5	74.4	70.5	62.3	-	Dick-Pérez et al., 2011
Xyloglucan	Terminal α-Xyl	99.9	80.9	74.7	70.2	62.0	-	York et al., 1993
Xyloglucan	Terminal α-Xyl (XgX^{t})	99.6	72.5	74.0	70.4	62.4	-	This work
Xyloglucan	Substituted α-Xyl (XgX^{S})	99.5	80.8	72.9	70.6	62.3	-	This work
Xyloglucan	Un-substituted Glc	105.0	72.7	75.9	83.0	73.8	61.5	Dick-Pérez et al., 2011
Xyloglucan	Substituted Glc	103.1	73.7	75.0	80.5	74.4	67.7	York et al., 1993
Xyloglucan	Un-substituted Glc (XgC^{u})	103.3	73.7	75.0	n/a	75.7	61.9	This work
Xyloglucan	Substituted Glc (XgC^{S})	103.3	73.7	75.0	79.8	74.0	67.0	This work
Xyloglucan	L-Fucose	100.7	n/a	n/a	n/a	n/a	17.2	Watt et al., 1999
Xyloglucan	L-Fucose	n/a	n/a	n/a	72.8	67.8	16.8	This work
Unknown	Terminal β-xylose	105.8	74.4	76.8	70.0	65.9	-	This work
Pectin	Galacturonic acid	99.3	69.0	72.9	n/a	n/a	n/a	This work
Arabinan	Terminal arabinose	108.4 /110.0	82.2	77.7	84.9	62.3	-	Wang et al., 2014
Arabinan	Internal arabinose	108.4 /110.0	82.2	77.8	82.5-83.2	64.0-67.9	-	Wang et al., 2014
Arabinan	Terminal arabinose (A^{t})	108.1 /107.6	81.9	77.4	84.8	61.9	-	This work
Arabinan	Internal arabinose (A^{c})	109.9	82.1	77.4	82.6	63.9	-	This work

Figures in black are literature assignments shown for comparison. Figures in blue are assignments from the current work. n/a: not assigned.

Supplemental Table S1. Chemical shifts (in ppm) for different moieties obtained with solution or solid state NMR (continued)

Solid State NMR------ ^{13}C NMR chemical shift of polysaccharides in WT callus								
Name	Type, moiety	C1	C2	C3	C4	C5	C6	References
Cellulose	Domain 1 (interior)	105.2	72.4	74.5	88.9	72.7	65.0	Dupree et al., 2015
	Domain 2 (surface)	105.2	72.4	74.5	84.2	75.5	62.7	
Cellulose	Interior (type a-e)	104.1-105.8	71.2-72.6	74.4-75.4	87.0-89.9	71.2-72.6	64.7-65.9	Wang et al., 2016
	Surface (type f, g)	104.9-105.1	72.5-72.9	74.1-75.3	83.5-84.5	75.3	61.5-62.5	
Cellulose	Domain 1 (C^1)	105.3	72.5	75.7	89.1	72.6	65.1	This work
		105.8	72.3	75.3	88.0	71.1	66.0	
		104.9	72.3	75.3	89.2	71.4	65.7	
	Domain 2 (C^2)	104.8	72.6	75.5	83.2	73.8	61.4	This work
		105.2	72.6	75.5	83.6	75.2	61.4	
		105.8	72.6	75.5	84.4	75.7	61.7	

Figures in black are literature assignments shown for comparison. Figures in blue are assignments from the current work. n/a: not assigned.

Supplemental Table S2. Plant species used for the phylogenetic tree

Plant Clade	Plant species
Charophytes	*Klebsormidium nitens*
Bryophytes	*Marchantia polymorpha, Physcomitrella patens, Sphagnum fallax*
Lycophytes	*Selaginella moellendorffii*
Polypodiopsa (ferns)	*Azolla filiculoides, Salvinia cucullata*
Gymnosperms:	*Cycas micholitzii, Ginkgo biloba, Gnetum montanum, Picea abies, Picea glauca, Picea sitchensis, Pinus pinaster, Pinus sylvestris, Pinus taeda, Pseudotsuga menziesii, Taxus baccata*
Basal angiosperms	*Amborella trichopoda, Nymphaea colorata, Liriodendron chinense*
Non-Poaceae monocots	*Ananas comosus, Apostasia shenzhenica, Asparagus officinalis, Calamus simplicifolia, Elaeis guineensis, Musa acuminate, Phalaenopsis equestris, Spirodela polyrhiza, Zostera marina*
Poaceae	*Brachypodium distachyon, Cenchrus americanus, Hordeum vulgare, Lolium perenne, Miscanthus sinensis, Oropetium thomaeum, Oryza brachyantha, Oryza sativa ssp. Indica, Oryza sativa ssp. Japonica, Phyllostachys edulis, Saccharum spontaneum, Setaria italica, Sorghum bicolor, Triticum aestivum, Triticum turgidum, Zea mays B73, Zea mays B104, Zea mays PH207, Zoysia japonica ssp. Nagirizaki*
Dicots	*Actinidia chinensis, Amaranthus hypochondriacus, Aquilegia caerulea, Arabidopsis lyrata, Arabidopsis thaliana, Arachis ipaensis, Beta vulgaris, Brassica oleracea, Brassica rapa, Cajanus cajan, Capsella rubella, Capsicum annuum, Carica papaya, Chenopodium quinoa, Cicer arietinum, Citrullus lanatus, Citrus clementina, Coffea canephora, Corchorus olitorius, Cucumis melo, Cucumis sativus L., Daucus carota, Erythranthe guttata, Eucalyptus grandis, Fragaria vesca, Glycine max, Gossypium raimondii, Hevea brasiliensis, Malus domestica, Manihot esculenta, Medicago truncatula, Nelumbo nucifera, Petunia axillaris, Populus trichocarpa, Prunus persica, Pyrus bretschneideri, Ricinus communis, Schrenkiella parvula, Solanum lycopersicum, Solanum tuberosum, Tarenaya hassleriana, Theobroma cacao, Trifolium pratense, Utricularia gibba, Vigna radiata var. radiata, Vitis vinifera, Ziziphus jujube*

Supplemental Table S3. Primers used in this study

Primer name	Sequence
Primers for identification of T-DNA insertion mutant	
SALK_065561-LP	CCCATCACTACCATCTTTCCC
SALK_065561-RP	TCATGCTCGAGGTGATATTCC
SAIL_852_F05C-LP	CCCATCACTACCATCTTTCCC
SAIL_852_F05C-RP	TCATGCTCGAGGTGATATTCC
SALK_065083-LP	AAAAAAGCATGTGTTGATGAACCC
SALK_065083-RP	GGTGGAAGTGGAGTGTCAAAG
SALK_071916-LP	CATTTTCACTAGATCCGCCAC
SALK_071916-RP	TCCGGTACAAGAACTGTAGCG
SALK_061576-LP	CAAGAACCAGACCGGTTGTATG
SALK_061576-RP	AGGTTGAAGAGGGGAAGTCTTG
SAIL_785_E02-LP	TAAACGTGTGTCCCCTAAACG
SAIL_785_E02-RP	AGAGAAATCTCGAGACCGGAC
SALK_101308-LP	TAAATTGTTTCCGCGGTACAC
SALK_101308-RP	AGTCACCAAAAGAACACGTGG
SALK_141953-LP	TGCAAACGAAATTAAACATAGGC
SALK_141953-RP	GAAGAAGAAACTGATTGGGGC
SALK_037323-LP	AAATAGGACGGTGGAGTGAG
SALK_037323-RP	ACTCTTGCATTCAGGGGAGG
MUR3-1-LP	CCAACCTACTTCCACCCAGC
MUR3-1 RP	GTTCTGATCTCGGGTCTGCG
GABI_552C10-LP	GGAAACCGCTCTTCCTACATC
GABI_552C10-RP	TCCGGTTTATCCGGTAAAAAC
Primers for amplification of MBGT1-3×Myc CDS	
MBGT1 forward	ATGCGACCCAAGAATTATTCTCAG
MBGT1 reverse	TCACAGATCTTCCTCAGAGA

Supplemental references:

1. **Dick-Pérez, M., Zhang, Y., Hayes, J., Salazar, A., Zabotina, O.A., and Hong, M.** (2011). Structure and interactions of plant cell-wall polysaccharides by two-and three-dimensional magic-angle-spinning solid-state NMR. Biochemistry-Us **50,** 989-1000.
2. **Schroder, R., Nicolas, P., Vincent, S.J., Fischer, M., Reymond, S., and Redgwell, R.J.** (2001). Purification and characterisation of a galactoglucomannan from kiwifruit (Actinidia deliciosa). Carbohydr Res **331,** 291-306.
3. **Terrett, O.M., Lyczakowski, J.J., Yu, L., Iuga, D., Franks, W.T., Brown, S.P., Dupree, R., and Dupree, P.** (2019). Molecular architecture of softwood revealed by solid-state NMR. Nat Commun **10,** 4978.
4. **Watt, D., Brasch, D., Larsen, D., and Melton, L.** (1999). Isolation, characterisation, and NMR study of xyloglucan from enzymatically depectinised and non-depectinised apple pomace. Carbohyd Polym **39,** 165-180.
5. **York, W.S., Harvey, L.K., Guillen, R., Albersheim, P., and Darvill, A.G.** (1993). The structure of plant-cell walls .36. Structural-analysis of tamarind seed xyloglucan oligosaccharides using beta-galactosidase digestion and spectroscopic methods. Carbohyd Res **248,** 285-301.
6. **Wang, T., Salazar, A., Zabotina, O.A., Hong, M.** (2014). Structure and dynamics of Brachypodium primary cell wall polysaccharides from two-dimensional ^{13}C solid-state nuclear magnetic resonance spectroscopy. Biochemistry **53**, 2840-2854.
7. **Dupree, R., Simmons, T.J., Mortimer, J.C., Patel, D., Iuga, D., Brown, S.P. Dupree, P.** (2015). Probing the molecular architecture of Arabidopsis thaliana secondary cell walls using two- and three-dimensional ^{13}C solid state nuclear magnetic resonance spectroscopy. Biochemistry **54**, 2335-2345.
8. **Wang, T., Yang, H., Kubicki, J.D., Hong, M.** (2016). Cellulose structural polymorphism in plant primary cell walls investigated by high-field 2D solid-state NMR spectroscopy and density functional theory calculations. Biomacromolecules **17**, 2210-2222.
