# Supplementary material for: Eudicot primary cell wall glucomannan is related in synthesis, structure, and function to xyloglucan
Source: Plant Cell. 2022 Aug 4;34(11):4600–22. doi: 10.1093/plcell/koac238 (PMC9614514; doi:10.1093/plcell/koac238)
Supplement: koac238_Supplementary_Data [file TPC2022RA00442R1_Peer_Review_Report.pdf › koac238_Supplementary_Data/TPC2022RA00442R1_Peer_Review_Report.pdf]

Eudicot primary cell wall glucomannan is related in synthesis, structure and function to xyloglucan

Li Yu, Yoshihisa Yoshimi, Rosalie Cresswell, Raymond Wightman, Jan J. Lyczakowski, Louis F.L. Wilson, Konan Ishida, Katherine Stott, Xiaolan Yu, Stephan Charalambous, Joel Wurman-Rodrich, Oliver M. Terrett, Steven P. Brown, Ray Dupree, Henry Temple, Kristian B.R.M. Krogh, Paul Dupree

Corresponding author: Paul Dupree pd101@cam.ac.uk

Review timeline:

**TPC2022-RA-00442**	Submission received:	May 11, 2022
	1st Decision:	June 14, 2022 *accept with minor revisions*
**TPC2022-RA-00442R1**	1st Revision received:	July 7, 2022
	2nd Decision:	July 14, 2022 *acceptance pending, sent to science editor*
	Final acceptance:	July 28, 2022

REPORT: (The report shows the major requests for revision and author responses. Minor comments for revision and miscellaneous correspondence are not included. The original format may not be reflected in this compilation, but the reviewer comments and author responses are not edited, except to correct minor typographical or spelling errors that could be a source of ambiguity.)

TPC2022-RA-00442 1st Editorial decision – *accept with minor revisions* **June 14, 2022**

On the basis of the advice received, the board of reviewing editors would like to accept your manuscript for publication in The Plant Cell. This acceptance is contingent on revision based on the comments of our reviewers. In particular, please consider the following:
Please respond to the specific comments raised by both reviewers and be sure examine the revisions suggested by reviewer 2

-- Reviewer comments:

Reviewer #1 (Comments for the Author):
The manuscript by Yu et al is a great contribution to science. This is another example of how cell wall related GT homologs are not redundant but have specific biochemical functions. A similar finding was published previously by the same group regarding xylan biosynthesis, and now we see a second example. The idea that glucomannans and xyloglucans have similar structures and biosynthesis and would support related functions in their interactions with cellulose is intriguing.
The data are well represented and the study is thorough. I only have two minor comments:
1. On pg 18, ln 362 it is stated that the csla2 mutant plants grew normally. That is not in agreement with Fig. 6. While the csla2 in the photos look very similar to WT, the graphs in panels C and E show that there are significant (albeit quantitatively small) differences.
2. The order of the legends in Fig. 5F is confusing. One would expect the order to be the same as the order of the columns, but they are not. mur3-3 and mbgt1-1/mur3-1 are swapped. This is a problem for colorblind persons who have to count from the left to know what bar represents what genotype. I needed help from another person to read this graph.
Reviewer #2 (Comments for the Author):
This is an impressive body of work. The manuscript presents nice data from well-designed and conducted experiments. Great work!
The reviewer has a handful of suggestions to improve this manuscript:
1) Various wording revisions to improve clarity

2) Adding more details, where indicated, in the Materials & Methods, to make techniques reproducible by others
3) Removing interpretation of results from figure legends and sticking with providing enough information to equip a

reader to interpret the data themself
4) I didn't understand why GMAB was selected for Figure 2F, since this oligosaccharide doesn't possess the alternating G-M backbone structure. Recommendation: use GBGA instead, since showing the structure could help a reader interpret Fig 2G
5) Insufficient evidence was provided to substantiate the claim that the proteins in Fig. 2E form stable dimers. If the authors have more evidence of this, it should be presented. At best, the data might suggest that the proteins form stable dimers.
6) It could be helpful for the authors to include a model figure at the end of the manuscript to compare/contrast structures/functions of XyG with bGGM in terms of interactions with cellulose, etc. This could really help put all the data into perspective]

TPC2022-RA-00442R1 1st Revision received July 7, 2022

Reviewer comments and **author responses**:

Reviewer #1 (Comments for the Author):
The manuscript by Yu et al is a great contribution to science. This is another example of how cell wall related GT homologs are not redundant but have specific biochemical functions. A similar finding was published previously by the same group regarding xylan biosynthesis, and now we see a second example. The idea that glucomannans and xyloglucans have similar structures and biosynthesis and would support related functions in their interactions with cellulose is intriguing.
The data are well represented and the study is thorough. I only have two minor comments:
1. On pg 18, ln 362 it is stated that the csla2 mutant plants grew normally. That is not in agreement with Fig. 6. While the csla2 in the photos look very similar to WT, the graphs in panels C and E show that there are significant (albeit quantitatively small) differences.

2. The order of the legends in Fig. 5F is confusing. One would expect the order to be the same as the order of the columns, but they are not. mur3-3 and mbgt1-1/mur3-1 are swapped. This is a problem for colorblind persons who have to count from the left to know what bar represents what genotype. I needed help from another person to read this graph.

We appreciate all these positive comments from Reviewer #1. We have revised both points by Reviewer #1.

As the reviewer mentioned, we acknowledge the minor but significant difference seen in plants height and silique length between *csla2* and WT. Therefore, we revised the sentence (line 358-359).

We swapped the positions of the *mur3-3* and *mbgt1 mur3-1* in Figure 6F accordingly. We apologise for the inconvenience.

Reviewer #2 (Comments for the Author):
This is an impressive body of work. The manuscript presents nice data from well-designed and conducted experiments. Great work!
The reviewer has a handful of suggestions to improve this manuscript:
1) Various wording revisions to improve clarity

2) Adding more details, where indicated, in the Materials & Methods, to make techniques reproducible by others
3) Removing interpretation of results from figure legends and sticking with providing enough information to equip a reader to interpret the data themself
4) I didn't understand why GMAB was selected for Figure 2F, since this oligosaccharide doesn't possess the alternating G-M backbone structure. Recommendation: use GBGA instead, since showing the structure could help a reader interpret Fig 2G
5) Insufficient evidence was provided to substantiate the claim that the proteins in Fig. 2E form stable dimers. If the authors have more evidence of this, it should be presented. At best, the data might suggest that the proteins form stable dimers.

6) It could be helpful for the authors to include a model figure at the end of the manuscript to compare/contrast structures/functions of XyG with bGGM in terms of interactions with cellulose, etc. This could really help put all the data into perspective

We appreciate the positive and constructive feedback given by Reviewer #2. We considered the reviewer's comments carefully and revised the manuscript. Following are the answers to the reviewer's points:

Thank you for suggesting the better wording. We accordingly revised the manuscript.

We revised the Materials & Methods accordingly.

According to the TPC guideline, it is encouraged to describe basic information necessary to understand the figures without referring the main text. Our figures are quite complex, so we believe that having such minimal interpretation information would help readers.

Thank you for the suggestion. We intended to put this to introduce the new codes for illustration of unsubstituted and substituted mannan structures rather than displaying an observed oligosaccharide. We understand that illustrating the oligosaccharides would be helpful to some extent for readers. A problem of using GBGA as an example here is to lose M for mannose without substitution from the figure, which we think is necessary. Thus, we decided to keep the one we have now.

As the reviewer pointed, we don't have strong or extra data supporting the statement of dimerization, which in any case is not an important point for the manuscript. So, we changed the statement in the legend to follow the suggestion.

Thank you very much for the suggestion. We have considered to include a summary model figure but at the moment we haven't fully revealed how and where (or which surface of cellulose) beta-GGM binds to cellulose, or how it compares to XyG. Additionally, the molecular function of both beta-GGM and XyG have not been elucidated completely. Therefore, we think that, in order to avoid a misleading over interpretation and conclusion for readers, it would be too early to draw a model including beta-GGM-cellulose binding and functions in cell elongation.

TPC2022-RA-00442R1 2nd Editorial decision – *acceptance pending* July 14, 2022

We are pleased to inform you that your paper entitled "Eudicot primary cell wall glucomannan is related in synthesis, structure and function to xyloglucan" has been accepted for publication in The Plant Cell, pending a final editorial review by a science editor. At this stage, your manuscript will be evaluated by a science editor with respect to its presentation of scientific content, compliance with journal policies, and presentation for a broad readership.

Final acceptance from Science Editor July 28, 2022
